# Sustained diabetes remission induced by FGF1 involves a shift in transcriptionally distinct AgRP neuron subpopulations

**DOI:** 10.1016/j.molmet.2025.102300

**Published:** 2025-12-09

**Authors:** Nadia N. Aalling, Petar V. Todorov, Shad Hassan, Dylan M. Belmont-Rausch, Oliver Pugerup Christensen, Claes Ottzen Laurentiussen, Anja M. Jørgensen, Kimberly M. Alonge, Jarrad M. Scarlett, Zaman Mirzadeh, Jenny M. Brown, Michael W. Schwartz, Tune H. Pers

**Affiliations:** 1 Novo Nordisk Foundation Center for Basic Metabolic Research, University of Copenhagen, Copenhagen, Denmark; 2 UW Medicine Diabetes Institute, Department of Medicine, University of Washington, Seattle, WA, USA; 3 McKnight Brain Institute, Department of Neuroscience, University of Florida, Gainesville, FL, USA; 4 Department of Neurosurgery, Barrow Neurological Institute, Phoenix, AZ, USA

**Keywords:** Fibroblast growth factor 1 (FGF1), Single nucleus RNA-seq, AgRP neuron heterogeneity, Diabetes, Perineuronal nets, Spatial transcriptomics

## Abstract

In rodent models of type 2 diabetes, a single intracerebroventricular (icv) injection of fibroblast growth factor 1 (FGF1) induces sustained remission of hyperglycemia. Overactive agouti-related peptide (AgRP) neurons, located in the hypothalamic arcuate nucleus, are a hallmark of diabetic states, and their long-term inhibition has been linked to FGF1's antidiabetic effects. To investigate the underlying mechanism(s), we performed single-nucleus RNA sequencing of the mediobasal hypothalamus at Days 5 and 14 post-injection in wild-type and diabetic (Lep^*ob/ob*^) mice treated with FGF1 or vehicle. We found that AgRP neurons from Lep^*ob/ob*^ mice form a transcriptionally distinct, hyperactive subpopulation. By Day 5, icv FGF1 induced a subset of these neurons to shift toward a less active, wild-type-like state, characterized by reduced activity-linked gene expression that persisted through Day 14. Spatial transcriptomics revealed that this FGF1-responsive AgRP subset is positioned dorsally within the arcuate nucleus. The transcriptional shift was accompanied by transcriptional processes indicative of increased GABAergic signaling, axonogenesis, and astrocyte–AgRP and oligodendrocyte–AgRP interactions. These glial inputs involve astrocytic neurexins and the perineuronal net (PNN) component phosphacan, suggesting both intrinsic and extrinsic mechanisms underlie FGF1-induced AgRP silencing. Combined with evidence that FGF1 increases PNN assembly in the arcuate nucleus, our findings reveal a cell-type–specific model for how FGF1 elicits long-term reprogramming of hypothalamic circuits to achieve diabetes remission.

## Highlights

• snRNA-seq finds a hyperactive, distinct AgRP neuron subtype in hyperglycemic leptin-deficient mice.
• Icv FGF1-induced diabetes remission drives in dorsal AgRP neurons a transcriptional state consistent with reduced activity.
• Reduced activity coincides with increased GABAergic signaling, axonogenesis, PNNs, and astro-/oligodendrocyte cues.

## 1 Introduction

Type 2 diabetes (T2D) is among the most common and costly diseases affecting Western society. Despite decades of study, the etiology of this disease remains poorly understood, with the consequence that many patients fail to reach glycemic goals [1], placing them at heightened risk of diabetes-associated complications. These considerations highlight the need to leverage an improved understanding of the disease into novel and more effective treatment strategies.

Growing evidence suggests that due to its privileged access to circulating signals through the median eminence and the 3rd ventricle, the mediobasal hypothalamus (MBH) plays a key role in glucose homeostasis [2]. Of particular interest is the arcuate nucleus-median eminence, situated along the floor of the 3rd ventricle, where several neuronal populations involved in glucose homeostasis are located. In 2016, we reported that a single intracerebroventricular (icv) injection of fibroblast growth factor 1 (FGF1) induces lasting normoglycemia in multiple rodent models of T2D [3], and in 2019, that FGF1 microinjection directly into the arcuate nucleus-median eminence recapitulates this sustained anti-diabetic effect [4]. A role for arcuate nucleus neurons in FGF1-induced diabetes remission was strengthened further by evidence that functional melanocortin 4 receptor (MC4R)-signaling is required for this effect [5]. In the arcuate nucleus, pro-opiomelanocortin (POMC) neurons activate, while agouti-related peptide (AgRP) neurons suppress, MC4R signaling in downstream neurons. These findings implicate AgRP and/or POMC neurons as likely mediators of the antidiabetic effect of FGF1, raising the possibility that FGF1 induces diabetes remission by inhibiting AgRP neurons, activating POMC neurons, or both. In support of this hypothesis, icv FGF1 injection induces a sustained downregulation of *Agrp* transcript levels in diabetic Lep^*ob/ob*^ mice [5], and also reduces the activity of at least some AgRP neurons for 14 days, based on electrophysiological recordings [6]. Strengthening this hypothesis is our recent finding that in the Lep^*ob/ob*^ mouse model of T2D, permanent AgRP neuron inactivation normalizes hyperglycemia for at least 10 weeks [7]. However, we are unaware of any precedent for how sustained reduction in activity of these neurons might be achieved.

In the current work, we used a combination of temporal single-nucleus (Days 5 and 14 post-injection icv) and spatial transcriptomics (Day 5) profiling to systematically *i)* map transcriptional heterogeneity within AgRP neurons in diabetic Lep^*ob/ob*^ mice and wild-type controls, *ii)* characterize the differences between these subpopulations and assess whether icv FGF1 injection induces a transcriptional shift in a subset of these neurons toward a wild-type-like state, *iii)* anatomically localize the FGF1-induced changes in Lep^ob/ob^ AgRP neurons, and *iv)* investigate cell signaling pathways from glial cells to AgRP neurons to shed light on potential mechanisms contributing to the sustained normoglycemic FGF1 effect.

We find that in diabetic Lep^*ob/ob*^ mice, AgRP neurons exhibit greatest rescue towards a wild-type phenotype than any other cell type five days after FGF1 injection, and that the FGF1-induced changes evolve to establish a transcriptional signature of reduced activity in a subset of Lep^*ob/ob*^ AgRP neurons by Day 14. The most prominent transcriptional programs increased in this FGF1-enriched subset include GABAergic receptor signaling and axonogenesis and we find that cells belonging to this subset are located furthest away from the ventral surface in the arcuate nucleus. We also identify enhancement of distinct signaling pathways from astrocytes and oligodendrocytes to AgRP neurons and report that following icv FGF1 injection, extracellular matrix specializations known as perineuronal nets (PNNs) are rapidly and robustly induced in the arcuate nucleus. Together, these findings point to *i*) changing interactions between glial cells, PNNs and a dorsally located subset of AgRP neurons, *ii*) transient increase in axonogenesis and GABAergic signaling gene transcription, and *iii*) sustained reduced AgRP activity, in the establishment and maintenance of sustained diabetes remission induced by icv FGF1 injection.

## 2 Materials and methods

### 2.1 Animals

Male Lep^ob/ob^(B6.V-Lep^ob^/JRj) and BL6 (C57BL6/JRj) from Janvier Labs, France, were housed at the University of Copenhagen, at 22 °C (+/−2 °C), with a 12/12 light/dark cycle (lights on at 7am) and with *ad libitum* access to water and standard chow food pellets unless otherwise stated. Mice were 11–12 weeks old and single-housed upon arrival. Food intake, blood glucose levels and body weight were measured in the mornings (between 9am and 12pm). Blood glucose levels were measured from a small incision of the tail vein with a Contour®XT apparatus using Contour®Next glucose strips (Ascensia Diabetes Care, Denmark). All experiments were performed in accordance with the Danish legislation approved by The Danish Animal Experiments Inspectorate (permit number 2019-15-0201-00073).

#### 2.1.1 ICV surgery & injection

A guide cannula (Plastics One, Roanoke, VA) was implanted to target the lateral ventricle using a stereotactic frame with the coordinates A/P:-0.7; L/V: 1.3; D/V: -1.3. Surgeries were performed under isoflurane anesthesia, lidocaine was injected to the incision line prior to incision of the skin and mice received Rimadyl (5 mg/kg) pre- and postoperatively. After one week of recovery, mice were injected with 1.5 μL of either recombinant human FGF1 (2 μg/μL, Novo Nordisk) or saline solution using a syringe pump at 2 μL/min. Four, five or 14 days after injection, mice were sacrificed by cervical dislocation.

#### 2.1.2 Experimental grouping and pair-feeding

Samples for snRNA-seq were generated from three batches of mouse experiments: i) Lep^ob/ob^ mice sacrificed 5 days after FGF1/saline injections (n = 4), ii) Lep^ob/ob^ mice sacrificed 14 days after FGF1/saline injections (n = 3), iii) Lep^ob/ob^ sacrificed 5 (n = 3) and 14 (n = 4) days after FGF1/saline injections, and BL6 mice sacrificed 5 (n = 4) and 14 (n = 4) days after saline injections. Samples for MC were gathered from one experiment. Lep^ob/ob^ mice were sacrificed 5 days after FGF1/saline injections (n = 4). Saline-injected mice were treated in parallel with FGF1-injected mice, receiving their injection one day later. Pair-feeding was implemented for the first 7 days following injections: food intake was recorded daily, and saline-treated mice were provided with the average amount consumed by FGF1-treated mice. After the 7-day period, all mice were allowed free access to food. Samples for histological detection of PNNs were gathered from an experiment wherein adult male Lep^ob/ob^ mice were sacrificed 4 daysd after icv injection of either FGF1 (3 μg) or saline vehicle (n = 5 saline, n = 4 FGF1) followed by cardiac perfusion using 0.1 M phosphate buffered saline (PBS) and fixation in 4% paraformaldehyde (PFA). Brains were cryopreserved in 30% sucrose prior to freezing in optimal cutting temperature (OCT) compound prior to sectioning on a Leica CM1950 cryostat at 30 um-thick serial sections and stored in 0.1 M PBS + 0.02% sodium azide.

#### 2.1.3 Statistical analysis

Statistical testing of differences between blood glucose, food intake and body weight of FGF1 and saline treated ob/ob mice after icv FGF1 administration (i.e. days 1–5 or 1–14) were performed using Graphpad Prism Two-way repeated measures ANOVA (based on GLM) with multiple comparisons when no overall effect of treatment, but an interaction between treatment and time were detected.

### 2.2 Immunohistochemistry

#### 2.2.1 Sample preparation and processing

30 μm-thick sections of free-floating tissues sections were processed for immunofluorescence according to previously published methods [8]. Free-floating brain sections were permeabilized for 30 min at room temperature in 0.1 M PBS + 0.2% Triton X-100 and blocked in 0.1 M PBS + 0.05% Triton X-100 (PBS-T) + 10% normal donkey serum (NDS) (Jackson ImmunoResearch Labs, Cat# 017-000-121) for 2 h at 37 °C. Sections were then incubated overnight at 4 °C using 1:1,000 dilution of biotin labeled *Wisteria floribunda* agglutinin (WFA; PNN CS-GAGs) (Sigma–Aldrich, Cat# L-1516) in PBS-T + 1% NDS. 16 h later, the sections were incubated for 2 h in 1:1,000 streptavidin-647 secondary antibody (ThermoFisher Scientific, Cat# S21374) in PBS-T + 1% NDS. Sections were mounted and cover slipped using Floromount-G (ThermoFisher, Cat# 4958-02).

Mean fluorescence intensity (MFI) of WFA was performed based on an established method for hypothalamic PNN quantification [8] and applied using a stereological approach. We first subdivided the hypothalamus into 5 Bregma positions (−1.6, −1.7, −1.8, −1.9, −2.0 mm from Bregma) defined based on the anatomical location of AgRP neural population of interest. From each defined region of interest, we used Fiji open-source imaging software to subtract a constant background and quantify the MFI (per mm^2^). We then computed the averaged normalized MFI by first normalizing MFI to the controls for each of the 5 regions separately and then averaging the normalized changes across all Bregma positions. Images taken on a Zeiss Axio Observer 7 inverted microscope with a 10x objective.

### 2.3 Single-nucleus RNA sequencing

#### 2.3.1 Sample preparation

##### 2.3.1.1 Tissue dissection

After cervical dislocation, brains were immediately removed from the skull, and one 1 mm section per brain was cut out using an ice cold adult mouse brain slicer matrix (BSMAS005-1, Zivic Instruments). The Arc-ME samples were dissected from the section by cutting a triangular piece off of the basal hypothalamus area. Samples were snap frozen on dry ice and stored at −80 °C until nuclei isolation.

##### 2.3.1.2 Nuclei isolation and sorting

Frozen Arc-ME samples were resuspended in an ice-cold Nuclei EZ lysis buffer (Sigma) and homogenized using a glass tissue douncer and pastel (Sigma). Homogenates were centrifuged (1,000 g, 5 min, 4 °C), the pellet resuspended in EZ lysis buffer, and the nuclei were filtered through a 40 μM cell strainer. After spinning down the nuclei (1000 g, 10min, 4 °C), they were resuspended and subsequently incubated in Nuclei buffer (PBS with 1% BSA, 2 mM MgCl_2_ and 40 U/μl Protector RNase inhibitor (Sigma)) on ice for 15 min. After being spun down again (1000 g, 10min, 4 °C), the pellet was resuspended in Nuclei buffer with 0,5 μg/μl TotalSeq-A Hashtag antibodies (Biolegend) and incubated on ice for 30 min. Cell hashing antibodies bind ubiquitously expressed surface proteins and thus uniquely label nuclei from individual samples. After adding the HashTag antibodies, nuclei were washed in Nuclei buffer, centrifuged (1000 g, 10min, 4 °C), and resuspended in Nuclei buffer with DAPI (0.5 μg/ml). Single DAPI^+^ nuclei were sorted on a SH800 cell sorter (Sony Biotechnology) with a 70 μm nozzle (Sony Biotechnologies, LE-C3207) directly into RT Reagent B from the Chromium NextGEM Single Cell 3’ kit v3.1 (10x Genomics).

Nuclei sorting and library preparation were done during three days for 12 samples per day. Each day contained samples mixed across groups to limit batch effects. Further batch effects were reduced by using cell hashing, which allows for sample pooling during sorting and subsequent RNA sequencing. When the Hashtags libraries are sequenced alongside the nuclei transcriptome, each nuclei can be assigned to its original sample.

Each day, 11500 nuclei were sorted into 5 wells in a 96-well plate (2300 nuclei per sample per well).

#### 2.3.2 Library preparation and RNA-sequencing

Single nucleus RNA-sequencing libraries were generated using the Chromium NextGEM Single Cell 3’ kit v3.1 (Dual Index) (10x Genomics) following the 10x User Guide in combination with a modified version of the TotalSeq™-A Antibodies and Cell Hashing with 10x Single Cell 3′ Reagent Kit v3.1 (Dual Index) Protocol (Biolegend). Briefly, the 10x protocol was followed until cDNA amplification, when 0.2 μM HashTag Oligonucleotide primer (TotalSeq™, Biolegend) was added to the cDNA amplification mastermix. After amplification, samples were mixed with SPRI beads binding the endogenous cDNA, while the supernatant containing HashTag cDNA were saved and used to generate the HashTag libraries in parallel with the nuclei libraries. The Qubit dsDNA HS kit was used on the Qubit 3 instrument (ThermoFischer Scientific) to quantify cDNA and libraries, and the TapeStation HS D1000 kit was used on the 4200 Tapestation (Agilent Technologies) to determine fragment size. The single nuclei libraries were sequenced on 3 x S2 flow cells using the Illumina NovaSeq 6000 platform following the NovaSeq 6000 Sequencing System Guide. Nuclei sorting, library preparation and RNA-sequencing was performed by the Single-Cell Omics platform at the Novo Nordisk Foundation Center for Basic Metabolic Research.

#### 2.3.3 snRNA-seq data processing & analysis

##### 2.3.3.1 Sequencing and preprocessing

BCL files were demultiplexed into FASTQ files using bcl2fastq v2.20.0.422.(*Bcl2fastq Conversion Software*, n.d.) Reads were pseudoaligned using Salmon Alevin v1.9.02 [9] with the flags *--read-geometry 2[1–15] --bc-geometry 1[1–16] --umi-geometry 1[17–26]* set. The RNA library was pseudoaligned to the GENCODE [10] vM23 reference transcriptome, distinguishing between spliced and unspliced transcripts. Alevin-fry [11] v0.7.03 was used to quantify both the RNA and hashtag oligonucleotide (HTO) libraries. Quantitated libraries were processed with Seurat [12]. Empty droplets were detected by the barcodeRanks function from the DropletUtils [13] package. HTOs were normalized using the *NormalizeData* function with the ‘CLR’ normalization method. A Gaussian mixture model with two components was fitted to each HTO distribution and a droplet was called to be positive for this HTO if it was predicted to belong to the cluster with the higher mean expression. Droplets positive for multiple HTO were classified as doublets. Inter HTO doublets were found using the *recoverDoublets* function form the scDblFinder package [14]. Cells negative for HTO library as well as doublets were removed from the pool at this stage. While the Salmon and Alevin-fry pseudocounts were used to classify droplets, we generated conventionally aligned counts via STAR using the CellRanger software, mapping to the GRCm38 mouse *Mus musculus* snRNA-seq genome supplied by 10x Genomics, Inc. We applied CellBender [15] to remove technical effects from the CellRanger count matrices using default parameters and the “remove-background” function.

#### 2.3.4 Normalization, dimensionality reduction, and clustering

To merge all three Illumina sequencing runs, we performed batch correction and data integration using a set of Seurat commands encapsulated in our process_seurat function. We utilized the FindTransferAnchors and IntegrateData functions with the sequencing run as the batch variable, using 15 PCA dimensions, 5000 features for anchor selection, 25 neighbors to discover anchors (k.anchor), 100 neighbors to weigh each anchor (k.weight), and a clustering resolution of 0.8 as default parameters.

For integrating subsets of the data in downstream analyses, optimal parameters needed to be selected in some instances. More narrow subsets representing cell type clusters could display batch effects at the level of each 10x microfluidics chip lane. As a result, we first attempted to regress out batch effects at the lane level, and if unsuccessful, we regressed out batches at the level of each Illumina sequencing run. In these instances, we tried k.weight values of 100, 50, 20, and 10 in descending order until the highest successful value resulting in batch removal (as determined by dimensional reduction plot inspection) was selected. The implementation of this approach is detailed in our accompanying code repository at https://github.com/perslab/Aalling-Todorov-Hassan-2025 and within the included target pipelines.

Following the above step, a total of 104,587 neuronal nuclei, and 29,782 nuclei from non-neuronal cell populations were retained. In non-neuronal cells, transcripts for a median number of 1308 genes, and a median number of 2336 transcripts per nucleus were detected. Expectedly, these numbers were slightly higher in neurons, where transcripts for a median number of 3269 genes and a median number of 7149 transcripts per nucleus were detected. In total, the analyses yielded seven non-neuronal cell clusters (>219 cells per cluster), and 45 neuronal cell clusters (>444 cells per cluster).

#### 2.3.5 Multi-level label transfer

First, the all cells in the integrated dataset were labelled using CellAnnotatoR [16] and a list of a priori set of mutually exclusive marker genes for neurons (expressed: *Rbfox3, Snap25*; not expressed: *Gfap, Aldh1l1, Aqp4, Bmpr1b, Fgfr3, Opalin, Plp1, Olig2, Olig1, Vcan, Gpr17, Cldn5, Pecam1, Rax, Col23a1, Crym, Lum, Col1a1, Pdgfrb, Foxj1, Pifo, Dynlrb2, Dcn, Cfh, Cped1*) and non-neurons. The dataset was then split into neurons and non-neurons which were each integrated separately using the 10x Chromium lane as a batch factor. CellAnnotatoR was used to label the non-neuronal subset based on predefined markers (See Supplementary Note 1). Neuron labels were transferred from a hypothalamic single cell atlas based on Affinati et al. [17] which was further refined using data from a number of scRNA-seq experiments in our lab. The reference object was subset to 20,000 cells to expedite computation time. Specific labels within the reference dataset were aggregated to broader categories to improve specificity and prediction accuracy. Cluster labels from the reference were transferred to the query object, and a prediction score was computed for each cell.

#### 2.3.6 Preparing objects for differential abundance testing

We used the miloR package to perform differential abundance (DA) testing within each cell type. After partitioning the dataset by cell type and normalizing it in Seurat, we added a metadata field (‘group’) representing the interaction of treatment, time, and strain. This variable defined experimental conditions for each cell. The Seurat object was converted to a SingleCellExperiment object for Milo compatibility. We built a k-nearest neighbor (kNN) graph using the top 15 principal components (PCs) and 40 nearest neighbors per cell. We then seeded transcriptional neighborhoods using makeNhoods (prop = 0.1, refined = TRUE), in which each seed cell and its 40 neighbors were refined to include the cell closest to the median PCA profile, reducing redundancy. Next, we used countCells to sum the number of cells from each experimental group per neighborhood, providing the basis for DA testing. Local neighborhood structure was quantified using calcNhoodDistance, which calculates Euclidean distances between cells in each neighborhood and informs FDR correction (SpatialFDR). Finally, we built a neighborhood–level graph using buildNhoodGraph, where nodes represent neighborhoods and edges reflect cell overlap.

#### 2.3.7 Differential abundance testing

A design dataframe was constructed. The metadata fields 'hash.mcl.ID', 'group', 'batch', and 'strain' representing individual cells, the combined group variable defined above, sequencing pool, and the mouse genotype, respectively, were converted to factors and included in the dataframe. The row names of the dataframe were set to correspond to the 'hash.mcl.ID' field, which uniquely identifies each animal. This design dataframe was used to model the experimental design in the differential abundance analysis. A model matrix taking into account the design and model ∼0 + group + batch was created. Differential abundance results were computed using the testNhoods function implemented in Milo. This function applies edgeR's QLF to the neighborhood counts, modeling the counts as a function of the experimental conditions specified in the design matrix. The QLF test provides a statistical measure of whether the abundance of cells in a neighborhood significantly changes across conditions specified by a given contrast. To account for differences in cell counts across neighborhoods, the Trimmed Mean of M-values normalization method was used. SpatialFDR correction adjusting for the total number of neighborhoods tested, as well as the Euclidean distance of their constituent cells was applied on a per-comparison basis.

#### 2.3.8 Labeling neighborhood states

Each neighborhood was assigned a polarity label using a custom function. Neighborhoods with a differential abundance log-fold_2_ change greater than zero and a SpatialFDR below 0.1 were labeled as “enriched", indicating a positive change in cell abundance. Conversely, neighborhoods with differential abundance log fold-change less than zero and a SpatialFDR below 0.1 were labeled as “depleted", indicating a negative change in cell abundance. Neighborhoods that did not meet these criteria were labeled as “none".

#### 2.3.9 Combining neighborhood state labels

Neighborhood labels resulting from the comparisons obob5v5 which indicates whether a neighborhood differential abundance is changed by FGF1 treatment in obob animals, and obobBL6 which determines whether differential abundance is changed due to genotype differences in vehicle treated animals were used to form a new combined label. The combined label resulting from the polarity outcomes for both contrasts ‘FGF1-ob *vs.* Veh-ob’ and ‘Veh-WT *vs.* Veh-ob’ are defined in Supplementary Note 2.

#### 2.3.10 Differential gene expression by neighborhood states

Neighborhood marker genes between distinct cellular states, as determined by the neighborhood-to-cell labeling procedure, were identified using the FindMarkers function in Seurat with the MAST method which allowed us to test for differential expression explicitly modelling animal ID as a latent variable. Each cellular label was treated as a distinct group, and marker genes were identified by comparing each group against one or all others, as specified (Supplementary Table 6). The function considered features present in at least 10 cells per group, with a minimum percentage of cells expressing the feature set at 0.01, and no log fold change threshold, thereby including both positive and negative markers. Additionally, we applied a heuristic cutoff at a minimum threshold of 250 cells per cluster when identifying neighborhood marker genes or supplying them into downstream analyses.

#### 2.3.11 Gene set enrichment analysis

Enrichment analysis was performed using the gost function from the gProfiler2 package [18]. For each set of genes (DEGs, WGCNA modules, etc), we performed an ordered query enrichment analysis against the Mus musculus genome namespace in all available databases, including Gene Ontology with electronic annotations. All p-values stated are adjusted at a false discovery rate of 0.05 using the g:SCS algorithm developed specifically for this approach. Only significant annotations are included in results.

#### 2.3.12 Single-cell WGCNA analysis

We performed a focused analysis on AgRP neurons in Lep^ob/ob^ animals. The dataset was filtered to include only cells from Lep^ob/ob^ animals at Day 5 and Day 14 and then preprocessed and normalized as described above. Single-cell data underwent preprocessing and analysis using the hdWGCNA R package [19,20,21]. Initially, cells were grouped based on unique identifiers such as animal ID, strain, and batch. Meta cells were then formed by aggregating counts from 25 cells selected from the kNN graph, with a restriction that no more than 15 cells could be shared between any two meta cells. The resulting meta cells were normalized, scaled, batch corrected using Harmony while modeling animal ID, and supplied for PCA and UMAP dimensional reduction using the built-in functions of hdWGCNA as described in the package vignettes with default parameters.

The expression matrix for hdWGCNA was instantiated using the scaled data RNA assay. A soft power threshold of 6 was selected by testing a range of integers between [1–30] and picking the first which reliably reached a scale-free topology fit above 0.8 and confirmed by visual inspection. The co-expression network was built, and module eigengenes, defined as the first principal component of the genes assigned to each module, were computed. Next Harmony batch correction was applied to mitigate technical variation while once again modeling each animal ID. The module hub genes were obtained by computing the eigengene-based connectivity (kME) determined by the correlation between the expression of the various genes and the module eigengene followed by selection of the top 25 genes by kME for each module. Differential module expression analysis was conducted using the FindDMEs function of hdWGCNA to compare FGF1-ob cells against Veh-ob cells and Veh-WT cells against Veh-ob cells. Detailed quality control and visualization steps were performed, including UMAP projections to inspect batch and strain effects on the resulting modules.

#### 2.3.13 CellChat analysis

The R Package *CellChat* version 2.1.2 [22] was used to identify up- and down-regulated signaling ligand-receptor pairs between the Day 5 FGF1-ob and Veh-ob snRNAseq datasets. Differential gene expression analysis using a Wilcoxon test with the presto package was performed using *identifyOverExpressedGenes()* with default parameters. After mapping the results to inferred cell–cell communications using *netMappingDEG(),* the up-/downregulated ligands (log_2_ fold-change = 0.1) and receptors (log_2_ fold-change = 0.2) were extracted using *subsetCommunication()*. Communication between glia cells (astrocytes, oligodendrocytes, OPCs, ependymal cells and tanycytes) as sources and AgRP neurons as targets were visualized using *netVisual_chord_gene()*.

### 2.4 Molecular cartography spatial transcriptomics

This section includes text provided by Resolve Biosciences describing the methods carried out while performing Molecular Cartography (MC) under contract for this study. Some segments are edited for conciseness or modified to reflect experiment-specific changes, while others are included verbatim.

#### 2.4.1 Probe design

A proprietary software algorithm (Resolve BioSciences GmbH, Monheim am Rhein, Germany) was deployed to design oligonucleotide probes targeting one hundred gene-level sequences. Full-length protein-coding transcripts from the ENSEMBL [23] database with the GENCODE 'basic' tag were used [10]. Jellyfish [24] was used to identify rare k-mers and de-prioritize repetitive regions which would not yield good probes. Probes were generated by extending rare k-mer regions until achieving target stability, applying heuristic to cut down suboptimal sequence candidates. Remaining candidates were mapped against the transcriptome with ThermonucleotideBLAST [25], and those scoring high for off-target hits were discarded. The scoring of probes considered on-target matches, weighted by APPRIS levels [26] biased towards selecting principal isoforms, with additional points for binding sites within protein-coding regions. The top-scoring probes were chosen to form the final set.

### 2.4.2 Tissue preparation

After cervical dislocation, brains were frozen immediately in powdered dry ice and stored at −80 °C until they were sectioned using a cryostat. Upon sectioning, tissue dorsal to and further lateral from the mediobasal brain were removed, and 10 μm thick sections of the mediobasal hypothalamus were cut and arranged in the capture sections of the pre-chilled Resolve Biosciences slides. Samples were packed in dry ice and shipped to Resolve BioSciences, where they were thawed on arrival and then fixed in 70% methanol diluted in 1x PBS for 20 min at 4 °C. Next, the sections were washed twice in 1x PBS for 1 min each, then washed for 1 min in isopropanol, followed by 100% ethanol and 70% ethanol, all at room temperature. Molecular Cartography was performed on fixed slides as follows. First, priming was performed for 30 min at 37 °C and subsequently targeted probes were hybridized to tissues overnight. Following the hybridization step, unbound probes were washed away from the samples, which were then fluorescently tagged by probes of two different colors. Designated areas of each sample were imaged, after which fluorescent signals were eliminated. The entire process was carried out recursively to interrogate the combinatorial barcode for each gene.

### 2.4.3 Imaging

Image acquisition was performed with a Zeiss Celldiscoverer 7 microscope equipped with a 50x Plan Apochromat water immersion objective (numerical aperture 1.2) and a 0.5x magnification changer, yielding an effective magnification of 25x. A standard LED excitation light source, filters, and dichroic mirrors provided illumination. Custom filters matched to signal fluorophores were used to filter incoming light. Probe detection was performed with an exposure time of 1000 ms per channel, while DAPI images were acquired with a 20 ms exposure time. Z-stacks were recorded for every region, with the distance between z-slices determined by the principles of the Nyquist-Shannon sampling theorem (Shannon, 1949) (Shannon, 1949) Practically, this means slice-to-slice distance is dynamically adjusted to be small enough to capture all the details in the sample without missing any important features. For example, if a sample has fine details or rapid intensity changes, it has high spatial frequency and requires smaller z-slice distances for accurate imaging.

A modified Zeiss Axiocam Mono 712 CMOS camera with a pixel size of 3.45 μm was used for image acquisition. For each region, a total of 16 z-stacks (two colors per imaging round, 8 rounds total) were acquired. A Python script interfacing with the Zeiss ZEN software API controlled the image acquisition process.

### 2.4.4 Preprocessing

To begin, all images’ background fluorescence was corrected. A target number of maxima was calculated by multiplying the slice area (in μm^2^) by an empirically optimized factor of 0.5. Maximum intensity peaks at a preset threshold were identified and recorded for each image slice. Maxima without neighboring maxima in adjacent slices (z-groups) were excluded. The remaining maxima were iteratively filtered based on brightness and background thresholds to reach a feature target value, with only maxima in z-groups of at least two being retained. Each cluster of point maxima was designated as a single hit, and the brightest maxima were saved as 3D-point cloud features.

Raw images from each round were aligned using extracted feature point clouds and an iterative closest point cloud algorithm [27,28] to minimize errors and segment probe signals. All point clouds were registered to the position of the first round's point cloud via trilinear interpolation. Pixels were filtered and grouped according to variance. Groups were filtered by size and adjacency, identifying local 3D-maxima as potential transcript locations, checked for fit to the probe code, and written to the results file as gene transcripts. Specificity was estimated by comparing signals matching experiment codes to those not used in the experiment.

### 2.4.5 Cell segmentation

Cell segmentation was performed using the Resolve Biosciences web interface and proprietary pipeline. To begin, MindaGap [29] was used to interpolate the gaps between imaging tiles in images and avoid incorrect segmentation. Following this, segmentation of cell nuclei in the processed image was obtained via the pre-trained cyto model included in Cellpose [30,31]. Segment diameter was set to 50.0 and image flow thresholdwas set to 0.5. Genes were designated as nucleus-specific if 70% or more of their transcripts are located within DAPI-stained segments, and as cytoplasmic otherwise.

CellPose DAPI segmentation is used as a prior for Baysor [16]. Baysor was invoked with the parameters: --no-ncv-estimation, --force-2d, --n-clusters = 1, and --prior-segmentation-confidence 0.9 -m 3. Nuclear segmentations from CellPose are used to identify nucleus- and cytoplasm-specific genes which are then supplied to Baysor via the --nuclei-genes and --cyto-genes parameters.

### 2.4.6 Format conversion of molecular cartography data to seurat v5

Due to a lack of official support from Resolve Biosciences for interoperability with Seurat, we developed resolve2xe [32] to convert the output from MC to emulate the 10x Xenium format which is supported by Seurat. This script first translates the Baysor results into a sparse matrix that includes the number of transcripts of each gene in every cell, with associated feature and barcode files. Then, it calculates the convex hull boundaries for each cell using the cell spatial coordinates. For Cellpose results, our script converts Resolve's ImageJ ROI files, which delineate the location of each nucleus within the cells, into a data frame format. We subsequently construct an R-tree data structure that can be used to rapidly map each nucleus or transcript to its containing cell based on spatial intersection with the cell's convex hull. Our script additionally generates cell statistics and transcripts data frames suitable for downstream analysis and visualization with Seurat's LoadXenium function.

### 2.4.7 Spatial transcriptomics preprocessing

**Spatial label transfer from snRNA-seq to MC data**.Spatial label transfer was performed using Robust Cell Type Decomposition (RCTD) [33] as implemented in the spacexr package. Unless specified otherwise, RCTD was executed in doublet mode with parameters: gene_cutoff = 10^−6^, fc_cutoff = 5 × 10^−2^, gene_cutoff_reg = 10^−6^, and a fc_cutoff_reg = 5 × 10^−2^. Cell class labels (neuron vs. non-neuron) were transferred from our single-nucleus RNA sequencing (snRNA-seq) dataset limited to Day 5 ob/ob samples as a reference. Labels were transferred to each tissue section individually.

Corrections to these labels were based on expression thresholds of key marker genes, which are specific to neurons and non-neurons. Briefly, expression of selected genes is assessed against predefined thresholds. The thresholds for gene-specific expression used to refine cell classifications were established heuristically. This was accomplished by qualitatively assessing the expression counts and distributions in cells, alongside their spatial plots. (data not shown) For instance, proteolipoprotein 1 (Plp1) is a component of the myelin sheath and a known oligodendrocyte marker, which we observed to be expressed in some cells initially labeled as neurons. To correct this, we established a threshold of 20 counts per cell for Plp1. Cells classified as neurons with Plp1 expression of 20 or more were reclassified as non-neuronal. Cells expressing Plp1 below 20 transcripts retained their neuronal classification. The same correction method was applied to reclassify cells initially labeled as neurons which expressed Pdgfra at a threshold of 10 transcripts. AgRP and Pomc were used for reclassifying non-neuronal cells as neurons at a threshold of 25 counts per cell.

After neuron and non-neuron cell classification was transferred onto cells, the dataset was split into these two subsets. RCTD was applied to transfer cell type labels to each slice individually, using the corresponding neuronal and non-neuronal subsets from our reference snRNA-seq dataset. In cases where RCTD failed to classify a cell, resulting in a “reject" status, these cells were also discarded. A similar approach of polarity transfer was performed on each cell type subset, excluding any “reject" cells.

Finally, all MC datasets were merged and processed using the steps outlined in the process_xenium function. Briefly, the default assay was set to 'Xenium', and a predefined set of 99 Xenium genes (excluding Lmx1a due to QC fail in Resolve's pipeline) was used. The data underwent normalization and scaling with default parameters, followed by PCA retaining the top 80 principal components. Subsequently, the data was clustered at a resolution of 2. A UMAP was constructed with the Xenium assay and counts slot directly.

The ɑ2 tanycytes label did not seem to have achieved optimal label transfer, whereas the ɑ1 tanycytes label covered most of tanycytes in the dorsal end of the third ventricle. The ɑ2 subtype was assigned to a small number of cells lining the outer edge of the brain along the ventral base of the hypothalamus.

### 2.4.8 Testing for between-cluster differences in MC data

To test whether the total number of cells in a given cluster changed between the FGF1 and Veh-ob groups, we deployed a generalized linear mixed-effects models using the glmer function from the lme4 package [34] in R. For each cell cluster, a new variable was created, categorizing cells as either belonging to the cluster of interest or to others. This variable was set as the response in a binomial generalized linear mixed-effects model, with treatment as the fixed effect and sample name as a random effect to account for within-sample variability. The p-values, coefficients, and z-scores were extracted directly from the models summaries. P-values were adjusted for multiple comparisons using the Benjamini-Hochberg method. A significance threshold of p_adj_ = 0.05 was used. This approach allowed us to assess the impact of treatment on the total number of cells in each cluster while accounting for multiple testing corrections.

### 2.4.9 Testing for within-cluster polarity differences in MC data

Spatial cells were tested for polarity changes (as assigned from milo label transfer). To evaluate the effect of treatment on the distribution of these classes, we chose a multinomial logistic regression model which is well-suited for predicting the outcome of a categorical variable (i.e. - polarity) with more than two levels. We used the multinom function implemented in the nnet R package to deploy the model [35]. For each label, we preprocessed the data by setting 'none' as the reference level for the polarity, with predictor variables including treatment and sample name. The data was then fit to model the probability of each category as a function of the predictors. Coefficients and their standard errors were extracted from the fitted model using the summary function, and z-values were computed by dividing each coefficient by its corresponding standard error. P-values derived from the z-values and adjusted for multiple comparisons using the Benjamini-Hochberg method. A significance threshold of *p*_adj_ = 0.05 was used.

In both of these instances we decided to further constrict the conditions under which a test could be significant. An additional filter requiring a minimum difference of 50 cells between the FGF1 and Veh-ob populations was put in place.

### 2.4.10 Proximity test for AgRP

The distance of the AgRP neurons (stratified by polarity) to the bottom of the ventricle was examined. For each section, the centroid of each AgRP polarity was calculated. The distance from each AgRP cell to the corresponding polarity's centroid was then computed, and cells with a distance greater than three standard deviations from the centroid were excluded as outliers. A pairwise comparison between the three polarities was then conducted. Within each section, the mean x-axis position was calculated for each polarity, and a paired t-test was performed for every polarity pairing using R's t.test function with the parameter paired = TRUE (pairings were defined within each section).

## 3 Results

### 3.1 Transcriptional response of AgRP neurons and other distinct hypothalamic cell populations five days after FGF1 administration

**Figure 1:**
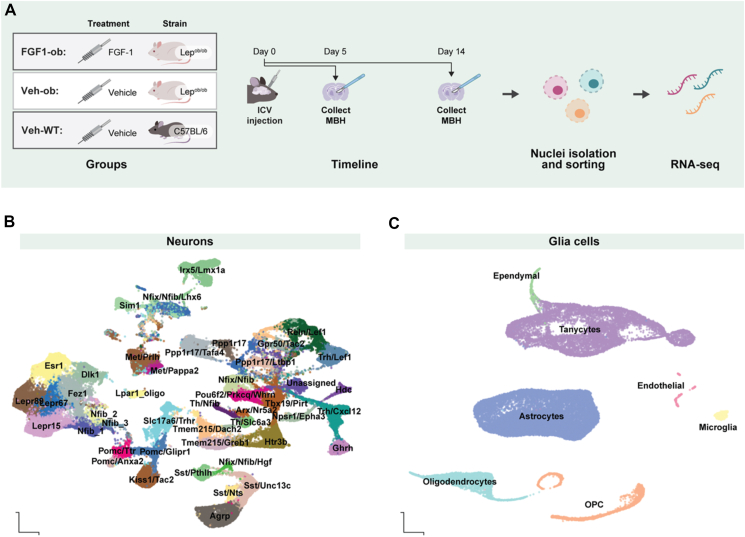
**snRNA-seq study design and identified cell populations**. **A**, FGF1 or saline was injected icv to Lep^*ob/ob*^ or C57BL/6 mice. BL6 and the group of Lep^*ob/ob*^ mice receiving saline were pair-fed to the FGF1 injected Lep^*ob/ob*^ mice (FGF1) for up to seven days after injection. Mediobasal hypothalamus samples for snRNA-seq were collected five or 14 days after injections and nuclei were sorted for snRNA-seq. **B,C,** UMAP plot of neuronal (left) and non-neuronal (right) cells across day five and 14 following label transfer from using data from Campbell et al. [38] and Affinati et al. [17]. Abbreviations: FGF1, fibroblast growth factor 1; icv, intracerebroventricular injection; UMAP, uniform manifold approximation and projection.

To determine the time course of the transcriptional effect of FGF1 on MBH cell types in a mouse model of T2D, diabetic Lep^ob/ob^ mice received an icv injection of either recombinant human FGF1 (3 μg; FGF1-ob) or saline vehicle (Veh-ob), while wild-type C57BL/6 control mice received icv vehicle (Veh-WT). Mice were euthanized either five or 14 days after icv injection and MBH harvested for analysis. Vehicle-treated groups (Veh-ob and Veh-WT) were pair-fed to the FGF1-ob group for the first 7 days after injection to ensure that blood glucose normalization was not attributable to reduced food intake (Figure 1A). Consistent with previous reports [[4], [5], [6],^36^,^37^], icv FGF1 injection normalized blood glucose levels in diabetic Lep^*ob/ob*^ mice independently of reduced food intake, such that blood glucose levels were significantly lower in FGF1-ob compared to Veh-ob animals (Supplementary Fig. 1). To characterize transcriptional cell profiles, we performed single-nucleus RNA-sequencing (snRNA-seq) on MBH tissue from each mouse. We applied nuclei isolation, nucleus multiplexing using hashtags, fluorescently activated nucleus sorting to reduce technical artifacts between groups, and retained 104,587 neuronal and 29,782 non-neuronal nuclei. Clustering identified 45 neuronal (Fig. 1B) and 7 non-neuronal populations (Fig. 1C), which were annotated using label propagation from published hypothalamic datasets [17,38].

**Figure 2:**
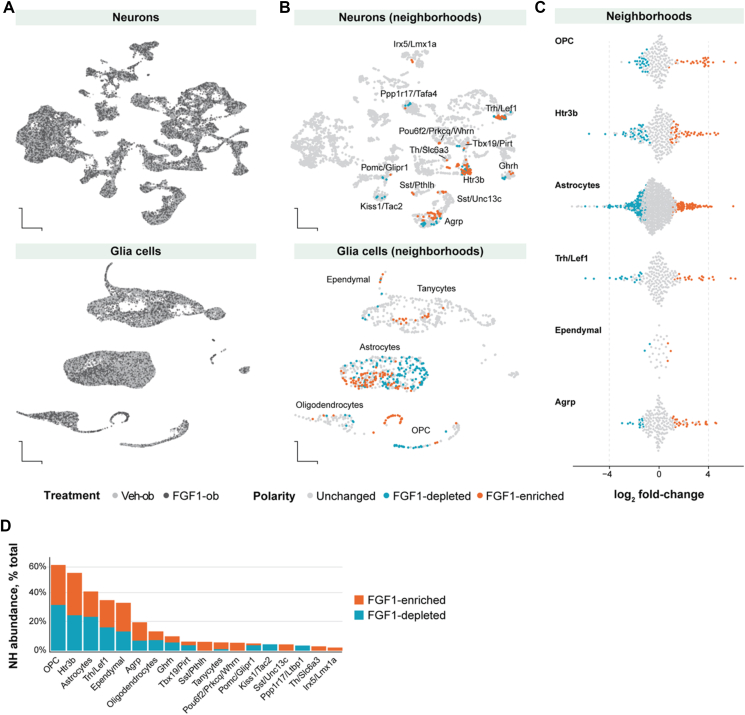
**FGF1 induces cell type-specific shifts in hypothalamic transcriptional neighborhoods at Day 5**. **A,** UMAP of all neurons highlighting cells from FGF1-ob (black) and Veh-ob (gray) mice. UMAP of all neuronal cells (upper panel) and non-neurons (lower panel) **B,** UMAP highlighting cells mapped to FGF1-enriched (red) and FGF1-depleted (blue) neighborhoods. **C**, Beeswarm plots of all neighborhoods across all cell populations with ≥20% regulated neighborhoods **D**, Percent FGF1-enriched (red) or FGF1-depleted (blue) neighborhoods out of the total number of neighborhoods in a given cell population Abbreviations: FGF1, fibroblast growth factor 1; icv, intracerebroventricular injection; UMAP, uniform manifold approximation and projection; FGF1-ob, Lep^*ob/ob*^ mice treated with FGF1 via icv injection; Veh-ob, Lep^*ob/ob*^ mice injected with saline and pair-fed to match food intake of FGF1-ob animals; Veh-WT, wild-type C57BL/6 mice injected with saline and pair-fed to match food intake of FGF1-ob animals; FGF1-depleted, neighborhoods in which the abundance of FGF1-ob cells is decreased relative to Veh-ob cells; FGF1-enriched, neighborhoods in which the abundance of FGF1-ob cells is increased relative to Veh-ob cells. (For interpretation of the references to color in this figure legend, the reader is referred to the Web version of this article.)

We next performed an unbiased transcriptomic analysis across all hypothalamic cell populations. Visualization of the snRNA-seq data revealed widespread transcriptional shifts between FGF1- and vehicle-treated diabetic Lep^*ob/ob*^ mice. As previously reported [5], the most robust transcriptional changes were observed in non-neuronal cells, but changes were also observed across many neuronal cell types (Fig. 2A). To systematically identify FGF1-induced transcriptional state changes within each population, we applied MiloR [39], a computational tool that detects abundance shifts in transcriptionally similar cell groups – termed 'neighborhoods' – revealing subtle intra-population treatment-induced changes. A neighborhood is considered FGF1-enriched if it contains a significantly higher proportion of cells from FGF1-ob mice and FGF-depleted if the opposite is true. Unlike analyses at the whole-population level, this approach more sensitively detects subsets of cells that exhibit coordinated transcriptomic changes across experimental groups and timepoints. Applying this approach, we identified significantly altered neighborhoods in 13 neuronal and five non-neuronal populations at Day 5 (Fig. 2B, Supplementary Fig. 2). In contrast, at Day 14 post-injection, only a small number of neighborhoods showed significant changes in cell abundance across all populations (Supplementary Fig. 3), highlighting the transient nature of early transcriptional responses to FGF1. Thus, while FGF1 induces robust and widespread transcriptional state changes within the first five days, these changes wane over time.

To rank the cell populations most affected by FGF1 at Day 5, we calculated the proportion of transcriptional neighborhoods with significant alterations in cell abundance relative to the total number of transcriptional neighborhoods within each population. Six cell populations exhibited substantial shifts, with at least 20% of neighborhoods affected (Fig. 2C). The most pronounced changes were observed in oligodendrocyte progenitor cells (OPCs, 60%), followed by Htr3b neurons (55%), astrocytes (42%), Trh/Lef1 neurons (35%), ependymal cells (33%), and AgRP neurons (20%). The cell populations showing the strongest response at Day 5 did not necessarily correspond to those with the highest expression of any of the FGF receptors known to be activated by FGF1 (Supplementary Fig. 4) [40], suggesting that indirect mechanisms or other factors may cause these changes. These findings confirm previously reported FGF1-induced transcriptional changes in several glial cell populations [5], while also uncovering shifts in three neuronal populations, most notably AgRP neurons (detailed below). Since these changes had largely disappeared by Day 14, the long-term metabolic effects of FGF1 are unlikely to be driven by persistent transcriptional remodeling. Instead, we hypothesize that early, temporally-restricted transcriptional changes initiate more stable adaptations affecting cell activity. Supporting data for this hypothesis is presented in the following sections.

### 3.2 FGF1-induced transcriptional shift in a subset of Lep^*ob/ob*^ AgRP neurons towards the wild-type subpopulation

**Figure 3:**
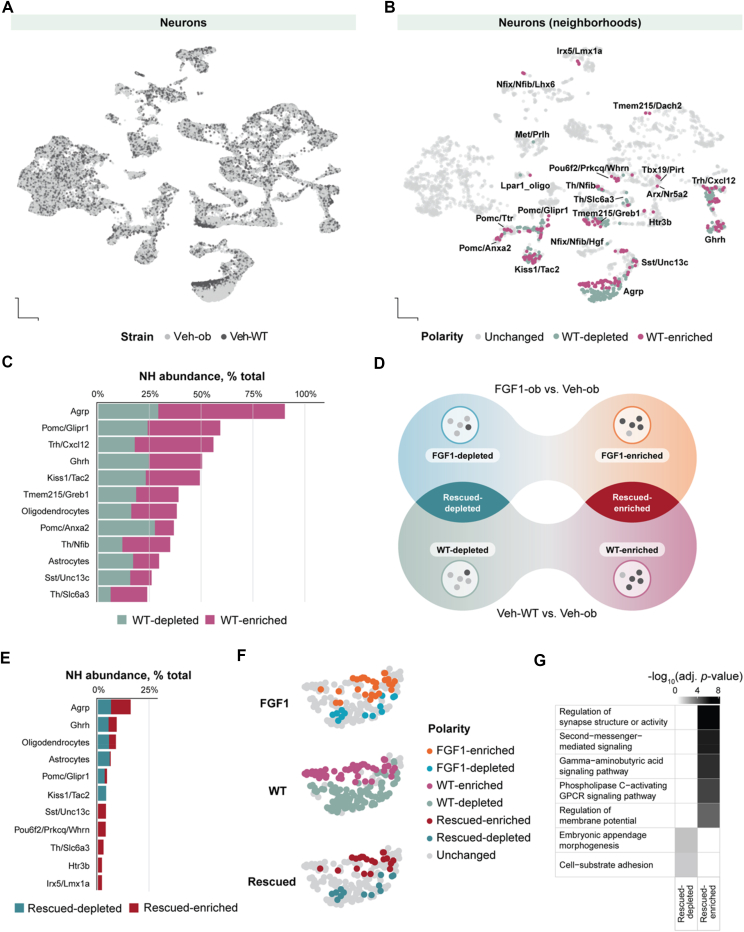
**Cell populations exhibiting transcriptional changes towards wild-type phenotypes five days after icv FGF1 injection**. **A,** UMAP of all neurons highlighting vehicle-treated cells across Lep^*ob/ob*^ and wild-type strains. **B**, UMAP of all neurons highlighting cells mapped to WT-enriched (magenta) and WT-depleted (green) neighborhoods. **C**, Percent WT-enriched or WT-depleted neighborhoods out of the total number of neighborhoods incell populations with at least 25% of NH abundance changed. **D**, Schematic of neighborhood abundance changes in FGF1 KO vs Veh-ob (top) and Veh-WT vs Veh-ob (bottom), illustrating FGF1- and WT-enriched/depleted neighborhoods. Central overlaps represent cells that change in the *same* direction in both analyses. **E,** Percent rescued-enriched or rescued-depleted neighborhoods out of the total number of neighborhoods in each cell population. **F**, Focused view of AgRP Milo neighborhoods in UMAP space. Each neighborhood is color-coded based on whether its cell abundance was significantly altered by icv FGF1 treatment, by genotype (Lep^*ob/ob*^*vs.* WT), and whether these changes reflect a rescue toward the wild-type state following FGF1 administration. **G**, Gene Ontology enrichment results depicting the most enriched terms for genes in depleted- and enriched rescued AgRP neighborhoods. Abbreviations: FGF1, fibroblast growth factor 1; icv, intracerebroventricular injection; UMAP, uniform manifold approximation and projection; FGF1-ob, hyperglycemic Lep^*ob/ob*^ treated with FGF1 via icv injection; Veh-ob, Lep^*ob/ob*^ mice injected with saline and pair-fed to match food intake of FGF1-ob animals; WT, wild-type animals injected with saline and pair-fed to match food intake of FGF1-ob animals; FGF1-enriched, neighborhoods in which the abundance of FGF1-ob cells is increased relative to Veh-ob cells; FGF1-depleted, neighborhoods in which the abundance of FGF1-ob cells is decreased relative to Veh-ob cells; WT-enriched, neighborhoods in which the abundance of WT cells is increased relative to Veh-ob cells; WT-depleted, neighborhoods in which the abundance of WT cells is decreased relative to Veh-ob cells; rescued-enriched, neighborhoods which register a significant differential abundance of FGF1-enriched and WT-enriched; rescued-depleted, neighborhoods that register a significant differential abundance of FGF1-depleted and WT-depleted. (For interpretation of the references to color in this figure legend, the reader is referred to the Web version of this article.)

Comparing UMAP visualizations of cells from Veh-WT to cells from diabetic Veh-ob mice (pair-fed to the reduced food intake of mice receiving icv FGF1) revealed pronounced differences across specific populations neurons (Fig. 3A). To detect and quantify subsets of cells transcriptionally differing between wild-type and mice, we identified neighborhoods in which the abundance of Veh-WT cells was either increased or decreased relative to Veh-ob cells. Among 23 cell populations with significant transcriptional alterations between vehicle-treated wild-type and Lep^*ob/ob*^mice at Day 5; 11 cell populations showed transcriptional shifts in more than 25% of their neighborhoods, underscoring the widespread impact of leptin deficiency on gene expression in mediobasal hypothalamic cells (Fig. 3B,C; Supplementary Fig. 2).

To assess whether FGF1-induced transcriptional changes resemble those observed in normoglycemic wild-type mice, we compared cell neighborhoods with altered cellular composition between FGF1- and vehicle-treated Lep^*ob/ob*^ mice (FGF1-ob *vs.* Veh-ob) and between wild-type and Lep^*ob/ob*^ vehicle-treated mice (Veh-WT *vs.* Veh-ob) at Day 5. Neighborhoods that exhibited significant compositional changes in both comparisons, and in the same direction, were classified as “rescued” by FGF1. Specifically, neighborhoods with increased cell proportions in both comparisons were defined as “rescued-enriched,” while those with decreased proportions were considered “rescued-depleted” (Fig. 3D).

At Day 5 transcriptional rescue was observed in 11 MBH cell populations, indicative of FGF1-mediated restoration toward a wild-type-like state (Fig. 3E). The highest proportions of rescued neighborhoods were observed in AgRP neurons (18%), Ghrh neurons (10%), and oligodendrocytes (10%). In these cell populations, a large fraction of the neighborhoods altered by FGF1 was changed toward a wild-type-like state: 100% of altered neighborhoods in Ghrh neurons, 90% in AgRP neurons, and 72% in oligodendrocytes resembled the wild-type transcriptional profile. These findings point to a strong restorative effect of FGF1 in these populations. By contrast, although OPCs, Htr3b neurons, and astrocytes exhibited strong transcriptional responses to FGF1 treatment (Fig. 2C,D), not all the responses in these populations recapitulated the transcriptional states observed in wild-type mice. Thus, these cell populations exhibited less FGF1-mediated rescue (Fig. 3E, Supplementary Fig. 2). Cells that exhibited significantly altered neighborhoods between Veh-WT and Veh-ob and were not rescued by FGF1 treatment included Trh/Cxcl12 and Tmem215/Greb1 neurons. Together, these findings highlight the selective and cell type-specific nature of FGF1's restorative effects, with certain populations, particularly AgRP and Ghrh neurons, and oligodendrocytes, undergoing robust transcriptional state rescue, while others, such as OPCs and Htr3b neurons exhibiting transcriptional responses to FGF1 that did not align with the wild-type profile.

Because AgRP neurons exhibited the highest proportion of rescued transcriptional neighborhoods following FGF1 treatment, we prioritized this population for further analysis.

FGF1 induced both rescue-enriched and rescue-depleted transcriptional responses in these neurons (Fig. 3F). Gene Ontology term enrichment analysis of genes upregulated in rescue-enriched transcriptional states revealed significant overrepresentation (*p*_*adj*_ *< 0.05*) of pathways related to “gamma-aminobutyric acid (GABA) signaling” and “regulation of synapse structure or activity,” including several GABA_A_ receptor subunit genes (e.g., *Gabra3, Gabra4, Gabra5*; Fig. 3G; Supplementary Table 1). These findings point to enhanced synaptic modulation and GABAergic responsiveness in AgRP neurons five days after FGF1 administration.

### 3.3 FGF1 drives early AgRP subpopulation shifts with increased GABA/axonogenesis and lasting suppression of activity-linked gene programs

**Figure 4:**
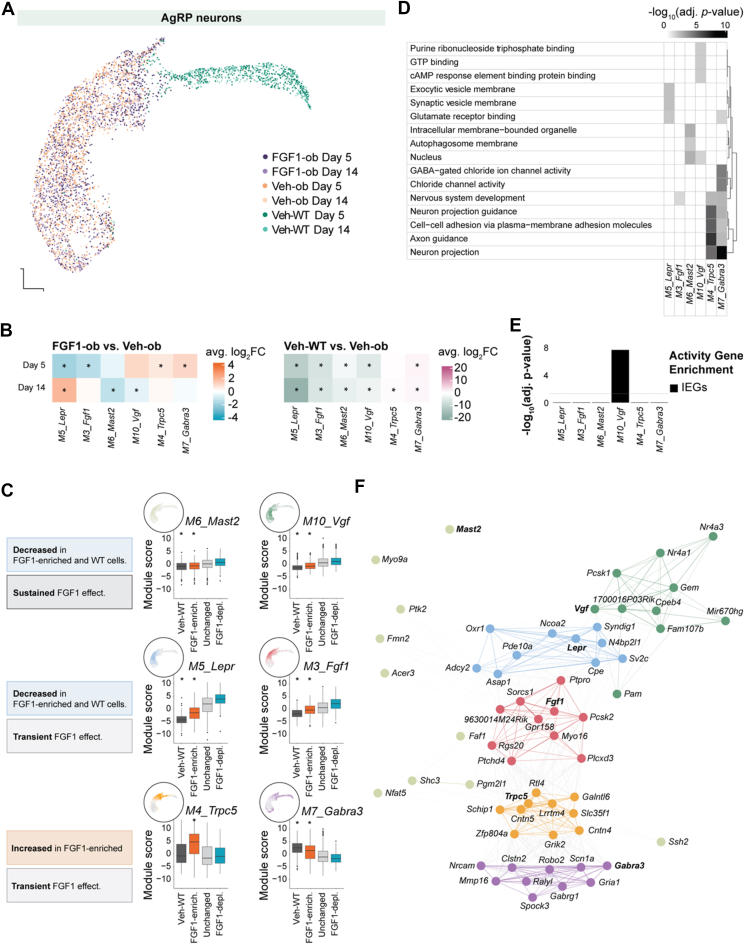
**Gene modules correlated with icv FGF1 injections at Days 5 and 14. A**, UMAP plot of AgRP neurons across Days 5 and 14 past icv FGF1 injection, across strains (Lep^*ob/ob*^, wild-type) and groups (FGF1-ob, Veh-ob, Veh-WT). Cells from Veh-WT animals differ strongly from cells from leptin-deficient animals (FGF1-ob, Veh-ob). **B**, Heatmap showing the results of differential module eigengene analysis for FGF1-ob *vs.* Veh-ob (left) and Veh-WT *vs.* Veh-ob (right). Colors are scaled by average log_2_ fold-change. Significantly different modules were annotated (∗, Bonferroni-adj. *p* < 0.05). **C**, UMAP and boxplot of module eigengene scores for the focal WGCNA modules; left UMAP panels show cells colored by their harmonized module eigengene score; boxplots compare the same eigengene scores across WT cells and all FGF1-polarized cells. Polarities significantly different from both FGF1-depleted and ‘Unchanged’ groups were annotated (∗, BH-adj. *p* < 0.05). **D,** Top 3 GO term enrichment heatmap for the focal WGCNA modules. **E,** One-sided Fisher's exact test for enrichment between module hub genes and neuronal activity IEGs. **F,** Combined module gene network plot (force-directed graph) for the focal WGCNA modules. Abbreviations: FGF1, fibroblast growth factor-1; icv., intracerebroventricular injection; UMAP, Uniform manifold approximation and projection for dimension reduction; FGF1-ob, hyperglycemic Lep^*ob/ob*^ treated with FGF1 via icv injection; Veh-ob, Lep^*ob/ob*^ mice injected with saline and pair-fed to match food intake of FGF1-ob animals; WT, wild-type animals injected with saline and pair-fed to match food intake of FGF1-ob animals; GO, gene ontology; BH, Benjamini-Hochberg. IEGs, immediate early genes. (For interpretation of the references to color in this figure legend, the reader is referred to the Web version of this article.)

To examine how FGF1 affects AgRP neuron function beyond early shifts, we analyzed their transcriptional regulation across both time points. Visualization of AgRP neurons harvested at five and 14 days after icv injections revealed that cells from Veh-WT vs. Lep^*ob/ob*^ (Veh-ob and FGF1-ob) mice occupied largely non-overlapping transcriptional spaces, highlighting a profound shift in AgRP transcriptome in diabetic, leptin-deficient mice (Fig. 4A). To further characterize the FGF1-induced changes in gene expression programs in AgRP neurons and their expression across these two distinct AgRP subpopulations, we applied Weighted Gene Co-expression Network Analysis (WGCNA). WGCNA is a method that identifies groups (or “modules”) of genes showing correlated expression patterns across cells, often reflecting shared biological functions [19,20]. In this analysis, each module represents a coordinated gene expression program potentially suggestive of specific cellular processes. We identified ten distinct gene co-expression modules across AgRP neurons from Veh-ob, and FGF1-ob mice at both Day 5 and Day 14 (Supplementary Fig. 5). Comparisons at Days 5 and 14 revealed seven gene modules significantly altered by FGF1 treatment, six of which also differed between Veh-WT and Veh-ob mice and were thus the focus of subsequent analyses (Supplementary Fig. 6; Fig. 4B).

We next asked whether the gene modules were significantly altered between WT AgRP neurons and the AgRP neurons in each of the three AgRP neighbourhoods/states identified above. The four modules that were downregulated in both the FGF1-enriched AgRP state and in wild-type AgRP neurons compared to the FGF1-depleted and unchanged AgRP states were *M3_Fgf1*, *M5_Lepr*, *M6_Mast2*, *M10_Vgf* (Fig. 4C). Module *M3_Fgf1* was enriched for ‘nervous system development’ and included the *Fgf1* gene, suggesting a compensatory decrease in *Fgf1* expression (Fig. 4D,F). The *M5_Lepr* module was enriched for the Gene Ontology terms ‘exocytic vesicle membrane’ and ‘glutamate receptor signaling’ and included the leptin receptor gene, *Lepr* in addition to *Sv2c* and *Cpe* encoding synaptic vesicle glycoprotein 2C and carboxypeptidase E, respectively (Fig. 4D,F; Supplementary Table 2). The shared decrease of these genes in WT and FGF1-enriched AgRP neurons is suggestive of a decreased responsiveness to leptin and a strong association of this with less neurotransmitter release and less synthesis of AgRP. These changes appeared transient, as expression levels of *M3_Fgf1* and *M5_Lepr* modules returned to baseline by Day 14 (Fig. 4B). In contrast, *M6_Mast2* and *M10_Vgf* remained suppressed in both FGF1-ob and Veh-WT compared to Veh-ob at Day 14. This pattern suggests that these two modules reflect baseline differences between diabetic leptin-deficient mice and Veh-WT mice that are restored by FGF1 and maintained over time. Five of the genes in *M10_Vgf* were immediate early genes (*Fos, Fosl2, Vgf, Nr4a1, Nr4a3*), and their expression levels are known to reflect the status of neuronal activity [41] (Supplementary Table 3). Further detailed analysis could verify that *M10_Vgf* enrich for immediate early genes (Fisher's exact test, *p* = 9 × 10^−5^; Fig. 4E) confirming to previous evidence that icv FGF1 injection induces a sustained decrease of AgRP neuron activity [6].

Two modules, *M4_Trpc5* and *M7_Gabra3*, were increased in the FGF1-enriched state at Day 5 (Fig. 4C). Module *M7_Gabra3,* which was also elevated in wild-type AgRP neurons, was enriched for several genes encoding different GABA_A_ receptor subunits suggesting increased responsiveness these rescued AgRP neurons to GABA, and additionally shared multiple Gene Ontology terms with *M4_Trpc5* including ‘neuron projection’ and ‘axon guidance’ suggesting an increased and rescue of axonogenesis after FGF1 injection. From these results we infer that a subset of AgRP neurons, the FGF1-enriched neurons, five days after icv FGF1 administration exhibits *i)* reduced expression of genes involved in neurotransmitter packaging, release, and leptin receptor signaling – processes indicative of lower neuronal activity, and *ii)* upregulation of axonogenesis and GABA_A_ receptor subunits genes, suggesting changes to the axonal projections and heightened GABA sensitivity. This transcriptional profile supports a model in which FGF1 reduces neuronal activity in a subset of AgRP neurons by changing it's projections and enhancing GABAergic inhibitory input, consistent with direct electrophysiological evidence in Lep^*ob/ob*^ mice following icv FGF1 injection [25].

### 3.4 A dorsal shift and glia-to-AgRP signaling support sustained decrease in AgRP activity

**Figure 5:**
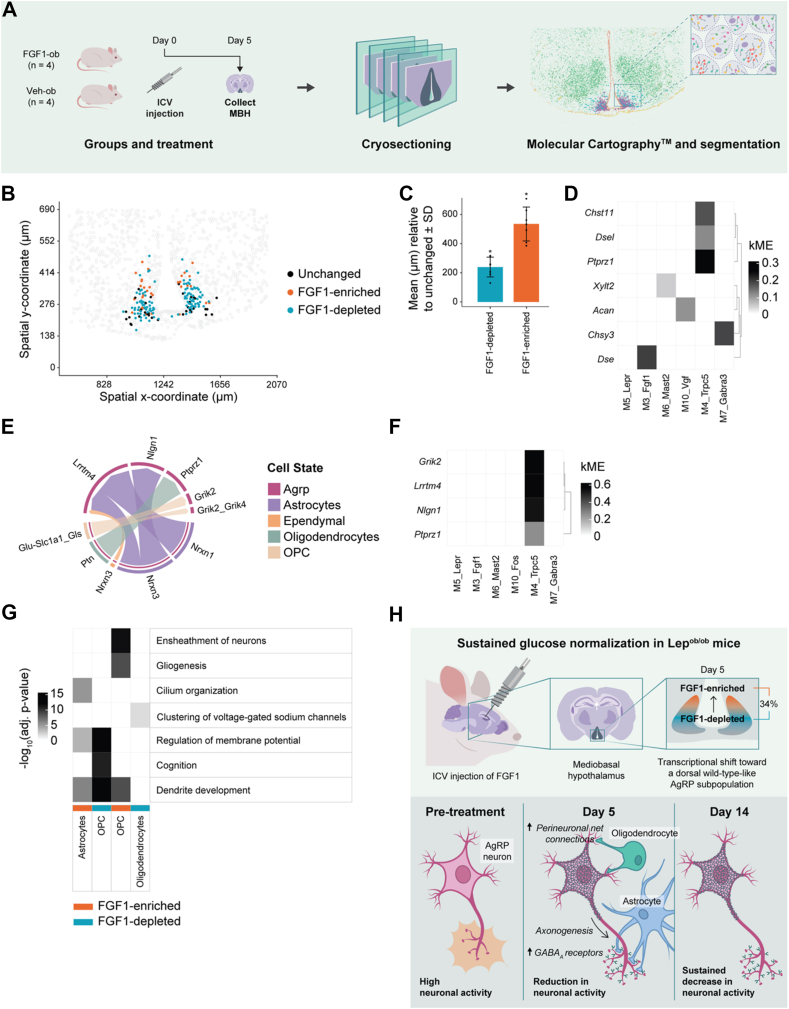
**Spatial mapping of transcriptomic changes following icv FGF1 injections**. **A**, Brains from four FGF1-ob and four Veh-ob mice were collected five days after injections and cryosectioned for Molecular Cartography. Cell segmentation was used to infer cell boundaries and map transcripts to cells. **B**, Spatial plot showing the bottom of the third ventricle in the hypothalamus. AgRP neurons with FGF1-depleted or FGF1-enriched polarity are color-coded accordingly. AgRP neurons with the ‘Unchanged’ annotation are coloured black, all other cells are coloured gray. **C**, Bar plot showing the mean vertical location of FGF1-depleted and FGF1-enriched AgRP neurons relative to ‘Unchanged’ across seven spatial transcriptomics sections. **D,** Heatmap showing the module assignment and module membership values (kME, eigengene-based connectivity) for perineuronal net genes. **E**, Chord diagram visualizing the cell–cell communication network of AgRP neurons and non-neuronal cells. **F,** Heatmap showing the module assignment and module membership values (kME, eigengene-based connectivity) for CellChat genes shown in E. **G**, Top 3 GO term enrichment heatmap for all available day 5 FGF1-polarities in astrocytes, OPC and oligodendrocytes. **H**, Following icv injection of FGF1, a subset of transcriptionally distinct, hyperactive AgRP neurons in Lep^*ob/ob*^ mice transcriptionally shifts toward a dorsal, wild-type-like state by Day 5. This transition is predicted to involve increased GABA_A_ receptor expression, axonogenesis, and enhanced glia–neuron interactions, including perineuronal net remodeling. By Day 14, AgRP neurons exhibit sustained suppression of activity-linked transcriptional programs, consistent with durable neuronal silencing and glycemic remission. Abbreviations: FGF1, fibroblast growth factor 1; UMAP, uniform manifold approximation and projection; icv, intracerebroventricular injection; FGF1-ob, hyperglycemic Lep^ob/ob^ treated with FGF1 via icv injection; Veh-ob, Lep^ob/ob^ mice injected with saline and pair-fed to match food intake of FGF1-ob animals; FGF1-depleted, neighborhoods in which the abundance of FGF1-ob cells is decreased relative to Veh-ob cells; FGF1-enriched, neghborhoods in which the abundance of FGF1-ob cells is increased relative to Veh-ob cells. (For interpretation of the references to color in this figure legend, the reader is referred to the Web version of this article.)

To further investigate the hypothalamic response to icv FGF1, we performed spatial transcriptomic profiling using Molecular Cartography on MBH sections collected from Lep^*ob/ob*^ mice five days after icv injection of FGF1 or vehicle (Fig. 5A). As in the previous experiment, vehicle-treated mice were pair-fed to the intake of FGF1-treated animals. FGF1 injection lowered blood glucose beginning on Day 4, with no major differences in food intake or body weight between groups (Supplementary Fig. 7). Around 28,000 high-quality cells from the Molecular Cartography data were segmented and annotated using a two-step pipeline combining DAPI-based segmentation (Baysor) with label transfer. Cell type and miloR-identified state labels were assigned based on the expression of 100 pre-selected transcript probes (Supplementary Table 4). Cell type assignments were confirmed by spatial localization of known marker genes, and key hypothalamic populations such as *AgRP*, *Pomc*, and β2 tanycytes (Supplementary Fig. 8).

To characterize the transcriptional AgRP states further, we mapped the spatial distribution of these on MBH sections from FGF1-ob and Veh-ob mice. A visual assessment indicated that the distribution of states was not uniform. We therefore measured their location along the ventral–dorsal axis of the arcuate nucleus in samples. Compared to AgRP neurons in the FGF1-unresponsive (‘unchanged’) state, AgRP neurons in both FGF1-enriched and FGF1-depleted states were positioned more dorsally, with the AgRP neurons in the FGF1-enriched state located furthest from the ventral surface (*p* < 0.001; Fig. 5B,C). A recent study has identified a dorsally located subpopulation of *Npy*-expressing neurons that are encased by the perineuronal net (PNN) proteoglycan aggrecan [42]. This subpopulation exhibited increased *Fos* expression, indicative of heightened activity, in comparison to all other AgRP neurons during fasting.

Given this finding, along with the established role of PNNs in mediating the long-term metabolic effects of FGF1 [41], we examined the association of PNN-related genes with the AgRP modules (Fig. 5D, Supplementary Table 5). Our analysis revealed that *Acan*, the gene encoding aggrecan, is part of the immediate-early gene module, *M10_Vgf*, which was suppressed by FGF1 at both Day 5 and Day 14. However, three other PNN-related genes were identified within the *M4_Trpc5* module*,* which is increased in the FGF1-enriched state at Day 5: *Ptprz1* (phosphacan), a chondroitin sulfate proteoglycan critical for PNN structure [43], alongside *Dsel* and *Chst11,* which are involved in the synthesis of chondroitin sulfate/dermatan glycosaminoglycans.

We next tested whether this transcriptional evidence suggestive of extracellular matrix remodelling associates with detectable changes in hypothalamic PNN content. To this end, we used *Wisteria floribunda* agglutinin (WFA) staining to compare hypothalamic PNN chondroitin sulfate/dermatan glycosaminoglycans abundance between FGF1-ob and Veh-ob mice. Tissue samples were collected 4 days after icv injection of either FGF1 (3 μg) or vehicle. Consistent with our transcriptomic findings, we report a 1.6-fold increase of WFA^+^ PNNs across the rostro-caudal extent of the arcuate nucleus in FGF1-ob compared to Veh-ob mice (Supplementary Figure 9). Collectively, these findings show that FGF1 changes PNN encoding genes in the dorsally located AgRP neuron subset which could influence the interaction of these AgRP neurons with PNN and PNN composition, particularly the chondroitin sulfate proteoglycan landscape.

Given the early and robust glial response to FGF1 observed previously [5], we investigated whether glial changes might contribute to sustained lowering of AgRP neuronal activity by performing ligand–receptor analysis using CellChat. Focusing on upregulated signals from glial cells to AgRP neurons at Day 5 after icv FGF1 injections, we identified two prominent glia-to-AgRP signaling pathways in FGF1-treated mice: *i)* astrocyte-derived neurexin (NRXN1 and NRXN3) signaling to NLGN1 and LRRTM4 receptors on AgRP neurons, and *ii)* oligodendrocyte-derived pleiotrophin (PTN) signaling to phosphacan (PTPRZ1) on AgRP neurons (Figure 5E; downregulated signaling results are found in Supplementary Fig. 10). These interactions align with the finding that astrocytes and oligodendrocytes are among the most transcriptionally responsive glial populations following FGF1 treatment (Fig. 2A–D). Notably, both *Lrrtm4* and *Ptprz1* are part of the *M4_Trpc5* gene module (Fig. 5F), which was elevated in the FGF1-enriched AgRP neuron subset on Day 5 (Fig. 4C). This supports our previous observation that FGF1 induces a neuron-interacting transcriptional state in astrocytes, along with a pronounced increase in astrocyte-AgRP neuron contacts [5]. Consistent with this, Gene Ontology analysis of FGF1-enriched astrocytes revealed significant enrichment for terms associated with synaptic interaction, including “regulation of membrane potential” and “dendrite development” (*p* < 0.05; Fig. 5G).

As previously shown [5], FGF1-treatment strongly affected the oligodendrocyte lineage (Fig. 2D) shifting OPCs towards a differentiating and more mature transcriptional phenotype as evidenced here by expression of markers such as *Bmp4* and *Mbp,* and enrichment for the Gene Ontology terms ‘Gliogenesis’ and ‘Ensheathment of neurons’ in FGF1-enriched OPCs (Fig. 5G, Supplementary Table 1). This FGF1-induced state shift in oligodendrocytes is further supported by the increased number of FGF1-enriched OPCs in MBH sections from FGF1-ob compared to Veh-ob mice (Supplementary Fig 8). OPC differentiation has previously been associated with PNN regulation in the MBH [44], and PNNs are required for the normoglycemic effect of FGF1 [45]. Thus, the identification of increased oligodendrocyte-derived pleiotrophin (PTN) signaling to the structural PNN component, phosphacan (PTPRZ1), on AgRP neurons on Day 5 suggests that early PNN remodeling could contribute to the persistent silencing of AgRP neurons following FGF1 treatment and potentially be driven by oligodendrocyte–AgRP interactions.

## 4 Discussion

Delineating the mechanisms by which a single icv injection of FGF1 leads to sustained diabetes remission in rodent models of T2D has implications for both basic and translational neuroendocrinology. While we and others have reported potent effects of FGF1 on gene expression, how these responses translate into durable effects on glycemia remained unclear. Here, we used single-nucleus and spatial transcriptomics to characterize hypothalamic responses to FGF1 in Lep^*ob/ob*^ mice, identifying molecular and cellular changes that offer a feasible and novel explanation for sustained diabetes remission. Our findings support a model in which an early shift in the composition of AgRP neuron subpopulations gives way to long-lasting changes that suppress the transcriptional activity of these neurons, potentially via both glia-mediated and cell-intrinsic mechanisms.

Comparing vehicle-treated Lep^*ob/ob*^ mice to WT controls reveals an AgRP neuron state in hyperglycemic Lep^*ob/ob*^ mice exhibiting activity-linked transcriptional features that are predicted to be associated with elevated baseline activity, which is well established in these animals [46]. Five days after icv FGF1 injection, the transcriptional phenotype of the AgRP neuron subpopulation in Lep^*ob/ob*^ mice shifted toward the phenotype of the wild-type subpopulation. This change coincided temporally with the normalization of glycemia and cannot be explained by differences of food intake or body weight, which were controlled for in the study design. Although this initial shift in subpopulation abundance had resolved by Day 14, transcriptomic profiling revealed sustained suppression of activity-linked gene modules in AgRP neurons. One module (*M10*_*Vgf*) showed significant enrichment for neuronal activity genes (e.g., *Fos, Vgf*) and remained downregulated at Day 14, consistent with the sustained decrease in AgRP neuronal activity after icv FGF1 treatment [7].

Using spatial transcriptomics, we mapped the distribution of FGF1-enriched, -depleted, and unchanged AgRP neuron states within the MBH of Lep^*ob/ob*^ mice. Our analysis revealed that FGF1-enriched AgRP neurons are predominantly localized to the most dorsal region of this population (Fig. 5B,C). Interestingly, recent work identified perineuronal nets (PNNs) surrounding a subpopulation of dorsal NPY neurons to be dynamically remodeled in response to metabolic state changes in mice [47]. During fasting, this remodeling involved a dorsal shift of the PNN-rich diffusion barrier between the median eminence and arcuate nucleus, facilitating access of blood-borne metabolic signals to more dorsally located arcuate neurons and augmenting their activity. Considering that increased PNN abundance can be a biological response mounted to constrain increased neuron activity [48], our finding of a robust increase of arcuate nucleus PNN content in FGF1-ob mice (Supplementary Fig. 10) offers a potential mechanism to explain FGF1's ability to limit AgRP neuron hyperactivity in these mice [49]. Additional studies are warranted, however, to ascertain whether dorsally-located AgRP neuron subpopulations showing reduced activity are among those that become enmeshed by PNNs following FGF1 administration.

Building on these observations, we speculate that the dorsal localization of FGF1-enriched AgRP neurons suggests a pivotal mechanism by which FGF1 mediates normoglycemia. Specifically, FGF1 may resensitize these neurons to blood-borne metabolic signals, thereby restoring their capacity to respond appropriately and dynamically to changes in systemic energy states. This reinstated responsiveness could facilitate downstream signaling critical for metabolic regulation. Further studies are needed to experimentally test this hypothesis and elucidate the underlying mechanisms.

Similar to our previous work [5], we here report robust transcriptional changes in multiple glial cells populations five days after icv FGF1 injection alongside the transcriptional changes observed in neurons, with most pronounced effects in OPCs and astrocytes. To identify how these changes in glia cells potentially could play into the subpopulation shift identified in AgRP neurons, we performed a ligand-receptor analyses to assess how outgoing signaling from all non-neuronal cells could interact with AgRP cells. This revealed enhanced signaling from astrocytes to AgRP neurons, particularly through neurexin-1 and -3 (NRXN1/3) binding to LRRTM4 and NLGN1 on AgRP neurons with FGF1 treatment. This finding extends earlier work showing that following icv FGF1 injection, the number of contacts between astrocytes and AgRP neurons increases markedly [5]. *Lrrtm4* and *Nlgn1* both central members of the *M4_Trpc5* module, were expressed at significantly higher levels in FGF1-enriched AgRP neurons compared to FGF1-depleted or wild-type AgRP neurons, confirming their specific induction by FGF1. Interestingly, LRRTM4, which localizes to GABAergic synapses, has been shown to promote GABA_A_ receptor clustering and enhance presynaptic inhibition [50]. These observations suggest that FGF1-induced astrocyte-AgRP interactions may play a role in regulating the expression of GABAergic receptors, specifically those in the *M7_Gabra3* module, which was restored by FGF1 treatment (Figure 3, Figure 4C). This regulation could contribute to the increased presynaptic inhibition previously reported in a subset (38.5%) of AgRP neurons following FGF1 application [6]. A second key glia-to-neuron interaction involved pleiotrophin from oligodendrocytes binding to phosphacan on AgRP neurons. In the hypothalamic arcuate nucleus, phosphacan a primary core protein contained in PNNs that enmesh AgRP neurons [51], and phosphacan is critical for the structure of PNNs as significant losses in the PNN marker wisteria floribunda agglutinin and in the PNN component aggrecan are seen in *Ptprz1* KO mice [43]. *Ptprz1* expression was enriched in the FGF1-enriched AgRP cells (*M4_Trpc5*) raising the interesting possibility that FGF1 via phosphacan increases the connectivity of the most dorsally located AgRP neurons with PNNs strengthening their structural integrity, which facilitates their reinstated responsiveness to blood-borne metabolic signals [19], [17]. Functional analysis of the FGF1-enriched *M4_Trpc5* AgRP module revealed enrichment of axonogenesis-related processes, including ‘Axon guidance’ and ‘Neuron projection’ (Fig. 4D). This finding is particularly intriguing in light of the defective development of AgRP neuron projections observed in Lep^*ob/ob*^ mice [53,54], suggesting that FGF1 may play a role in restoring or compensating for failed downstream projections from AgRP neurons.

While AgRP neurons showed the strongest transcriptional rescue toward wild-type states, Htr3b and Ghrh neurons were the cell populations exhibiting the most pronounced transcriptional responses to icv FGF1 administration and the highest rescue percentage, respectively. Htr3b neurons in the MBH have previously been shown to be glucose-excited [55], while Ghrh neurons are activated by hypoglycemia [56] and based on unpublished transcriptional data, inhibited by hyperglycemia (Brown et al., *submitted*). Additional studies are needed to determine how icv FGF1 affects these neurons *in vivo* and whether their transcriptional remodeling contributes to the maintenance of diabetes remission. Taken together, our findings support a model in which astrocytes and oligodendrocytes contribute to the sustained reduction in AgRP neuron activity through complementary mechanisms; astrocytes by enhancing inhibitory synaptic input and oligodendrocytes by promoting PNN connections. These glia–neuron interactions appear to be initiated shortly after FGF1 injection and may provide the structural and molecular framework for maintaining reduced AgRP neuron activity beyond the transient shift in transcriptional subpopulations, ultimately contributing to sustained diabetes remission (Fig. 5H).

Using unbiased transcriptomic techniques at single-cell resolution at two distinct timepoints following an icv injection of FGF1, this study not only corroborated findings from earlier investigations into the effects of a single icv FGF1 injection on the MBH but also extended these results. Specifically, it provided novel functional, cellular, and molecular insights, culminating in a testable model for how FGF1 induces normoglycemia. Future work should determine whether the identified FGF1-induced transcriptional changes specifically in dorsally located AgRP neurons are necessary and sufficient for sustained normalization of blood glucose. Moreover, additional studies are needed to clarify the specific contributions of the identified interactions between this AgRP subset and astrocytes and oligodendrocytes, and whether these interactions are required for the changes in AgRP neurons as hypothesized here.

## CRediT authorship contribution statement

**Nadia N. Aalling:** Writing – review & editing, Writing – original draft, Visualization, Methodology, Investigation, Formal analysis, Data curation, Conceptualization. **Petar V. Todorov:** Writing – original draft, Software, Resources, Methodology, Investigation, Formal analysis, Data curation. **Shad Hassan:** Writing – review & editing, Software, Methodology, Investigation, Formal analysis, Data curation. **Dylan M. Belmont-Rausch:** Writing – review & editing, Investigation, Formal analysis. **Oliver Pugerup Christensen:** Writing – review & editing, Methodology, Formal analysis. **Claes Ottzen Laurentiussen:** Writing – review & editing, Methodology, Formal analysis. **Anja M. Jørgensen:** Writing – review & editing, Formal analysis. **Jenny M. Brown:** Writing – review & editing, Investigation, Formal analysis. **Michael W. Schwartz:** Writing – review & editing, Investigation. **Tune H. Pers:** Writing – review & editing, Writing – original draft, Supervision, Resources, Project administration, Investigation, Funding acquisition, Conceptualization. **Zaman Mirzadeh:** Writing - review & editing, Resources. **Jarrad M. Scarlett:** Writing – review & editing, Resources. **Kimberly M. Alonge:** Writing – review & editing, Resources, Formal Analysis.

## Declaration of generative AI and AI-assisted technologies in the writing process

During the preparation of this work the author used ChatGPT in order to improve the readability and language of the manuscript. After using this tool/service, the author reviewed and edited the content as needed and take full responsibility for the content of the published article.

## Declaration of competing interest

The authors declare the following financial interests/personal relationships which may be considered as potential competing interests: Nadia Aalling reports a relationship with Novo Nordisk that includes: employment. Dylan M. Belmont-Rausch reports a relationship with Novo Nordisk that includes: employment. Tune H Pers reports a relationship with Novo Nordisk that includes: equity or stocks. If there are other authors, they declare that they have no known competing financial interests or personal relationships that could have appeared to influence the work reported in this paper.

## Data Availability

All code is available at https://github.com/perslab/Aalling-Todorov-Hassan-2025.
