# Supplementary material for: Sustained diabetes remission induced by FGF1 involves a shift in transcriptionally distinct AgRP neuron subpopulations
Source: Mol Metab. 2025 Dec 9;103:102300. doi: 10.1016/j.molmet.2025.102300 (PMC12808567; doi:10.1016/j.molmet.2025.102300)
Supplement: Multimedia component 2 [file mmc2.docx]

# Supplementary Figures


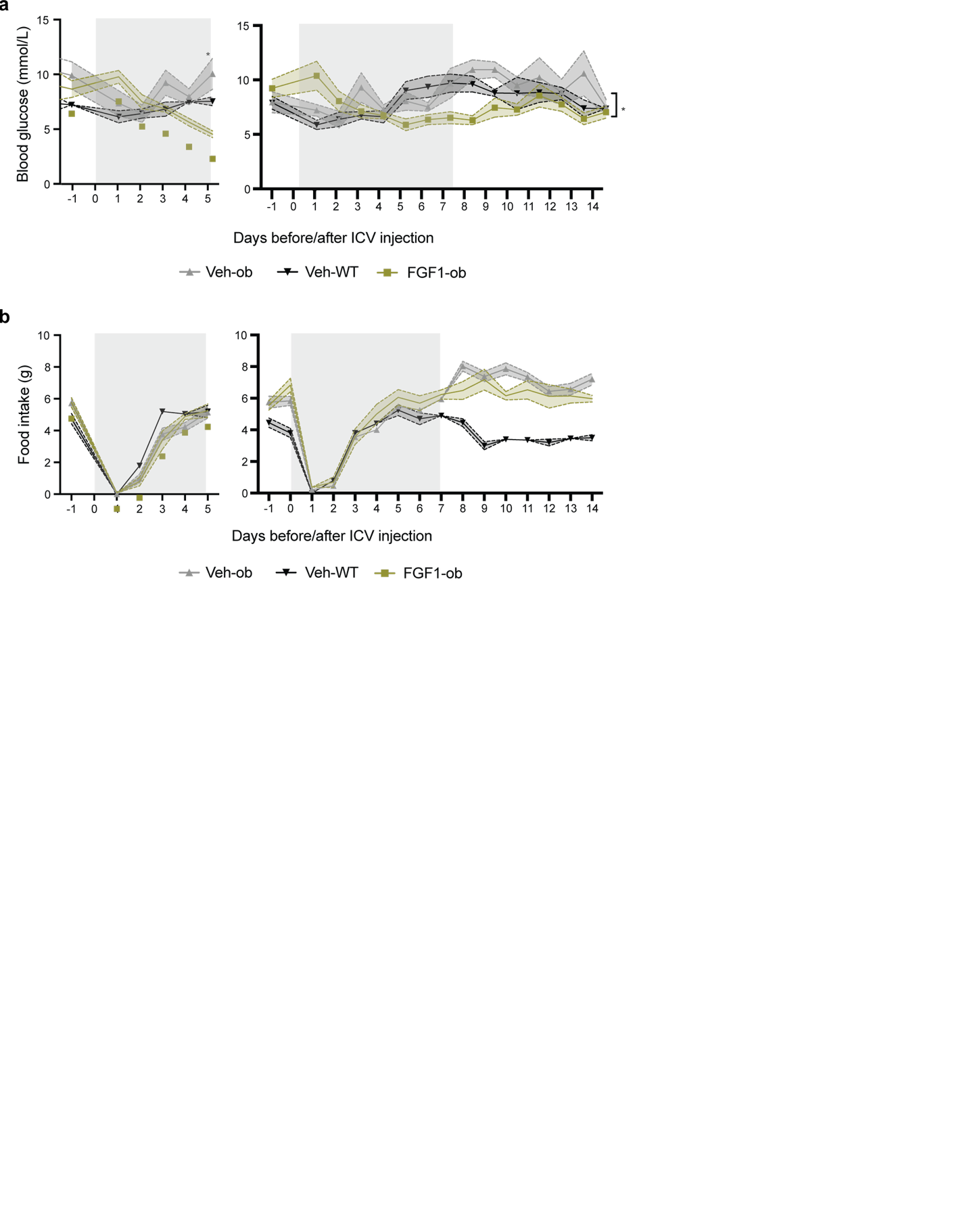


**Supplementary Figure 1**. **a**, Blood glucose measurements of FGF1-ob, Veh-ob, or Veh-WT-group mice sacrificed five or 14 days after injections. **b**, Food intake measurements of FGF1-ob, Veh-ob, or Veh-WT-group mice sacrificed five or 14 days after injections. Differences between groups were identified using two-way ANOVA with multiple comparisons when applicable (*, p<0.05). Abbreviations: FGF1, fibroblast growth factor 1; FGF1-ob, Lep^ob/ob^ mice treated with FGF1 via icv injection; Veh-ob, Lep^ob/ob^ mice injected with saline and pair-fed to match food intake of FGF1-ob animals; Veh-WT, wildtype C57BL/6 mice injected with saline and pair-fed to match food intake of FGF1-ob animals; ANOVA, analysis of variance


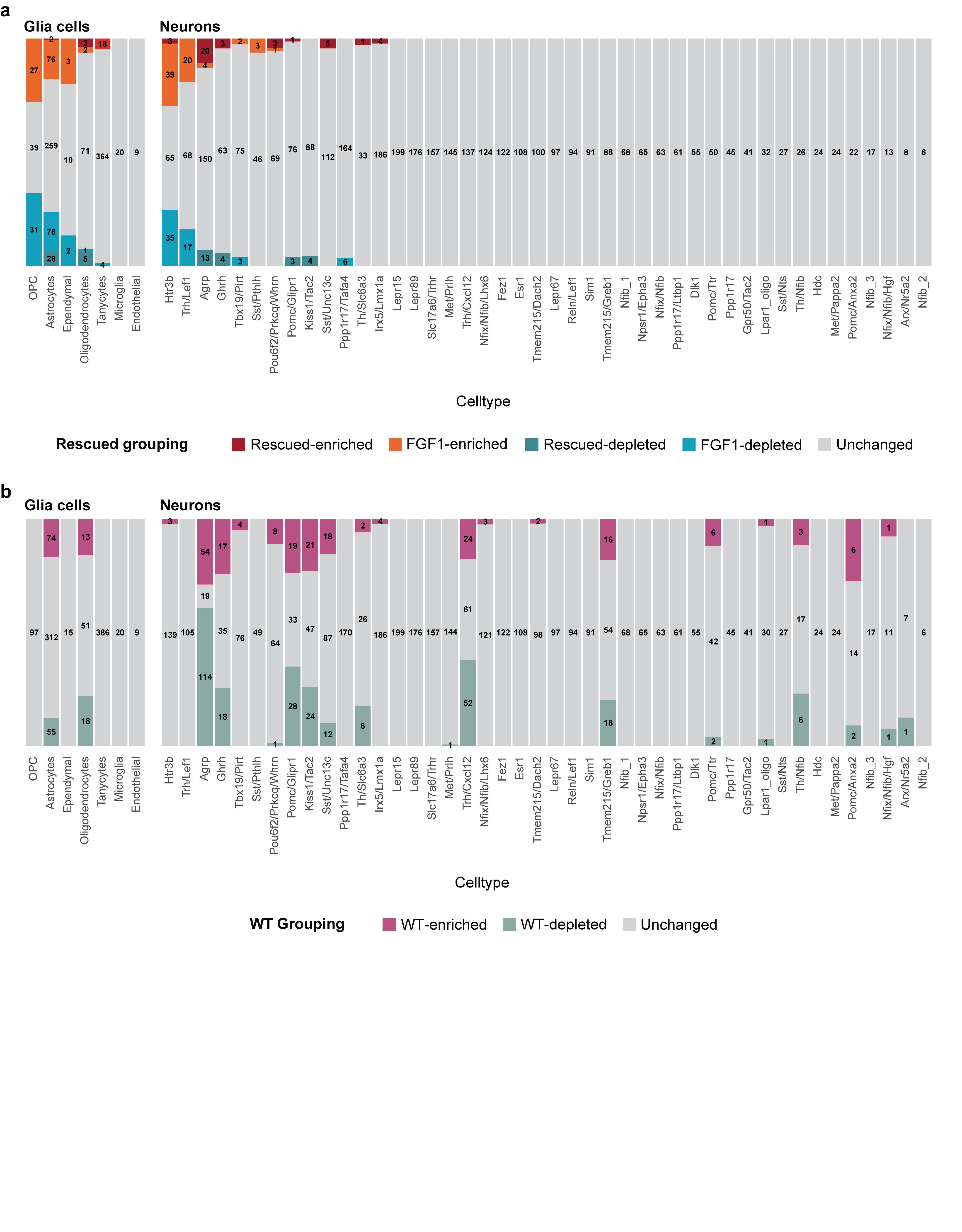


**Supplementary Figure 2**. **a**, Number of neighborhoods in glia and neurons that are FGF1-enriched, FGF1-depleted, rescued-enriched, rescued-depleted, or unchanged, out of the total number of neighborhoods in glia cells and neurons. Analysis was carried out on day 5 FGF1-ob vs. Veh-ob cells. **b**, Number of neighborhoods in glia and neurons that are WT-enriched, WT-depleted, or unchanged, out of the total number of neighborhoods in glia cells and neurons. Analysis was carried out on day 5 Veh-ob vs. Veh-WT cells. Abbreviations: FGF1, fibroblast growth factor 1; FGF1-ob, Lep^ob/ob^ mice treated with FGF1 via icv injection; Veh-ob, Lep^ob/ob^ mice injected with saline and pair-fed to match food intake of FGF1-ob animals; Veh-WT, wildtype C57BL/6 mice injected with saline and pair-fed to match food intake of FGF1-ob animals; FGF1-depleted, neighborhoods in which the abundance of FGF1-ob cells is decreased relative to Veh-ob cells; FGF1-enriched, neighborhoods in which the abundance of FGF1-ob cells is increased relative to Veh-ob cells; WT-depleted, neighborhoods in which the abundance of WT cells is decreased relative to Veh-ob cells; WT-enriched, neighborhoods in which the abundance of WT cells is increased relative to Veh-ob cells; rescued-depleted, neighborhoods which register a significant differential abundance of FGF1-depleted and WT-depleted; rescued-enriched, neighborhoods that register a significant differential abundance of FGF1-enriched and WT-enriched.


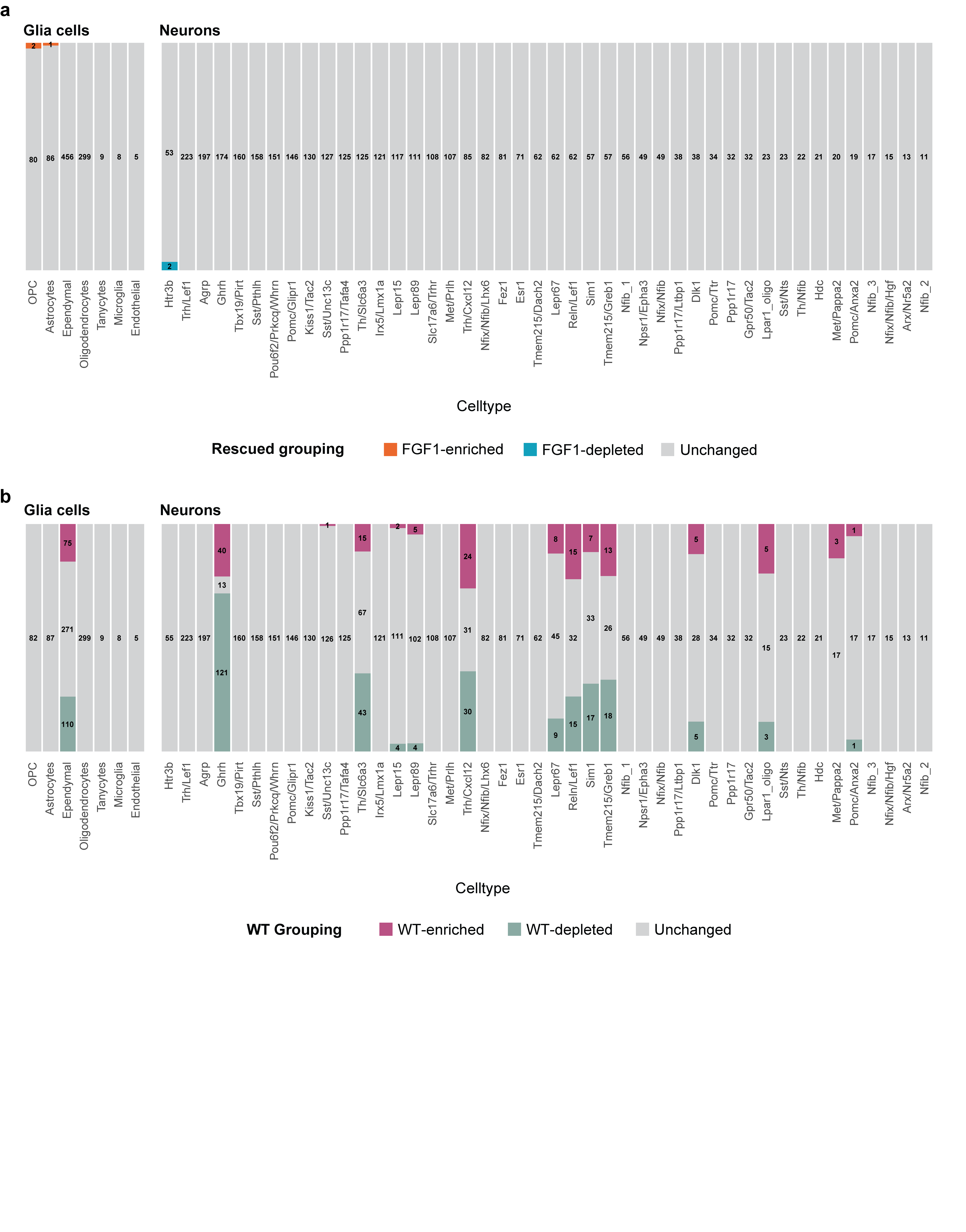


**Supplementary Figure 3**. **a**, Number of neighborhoods in glia and neurons that are FGF1-enriched, FGF1-depleted, rescued-enriched, rescued-depleted, or unchanged, out of the total number of neighborhoods in glia cells and neurons. Analysis was carried out on day 14 FGF1-ob vs. Veh-ob cells. No rescued neighborhoods were identified. **b**, Number of neighborhoods in glia and neurons that are WT-enriched, WT-depleted, or unchanged, out of the total number of neighborhoods in glia cells and neurons. Analysis was carried out on day 14 Veh-ob vs. Veh-WT cells. Abbreviations: FGF1, fibroblast growth factor 1; FGF1-ob, Lep^ob/ob^ mice treated with FGF1 via icv injection; Veh-ob, Lep^ob/ob^ mice injected with saline and pair-fed to match food intake of FGF1-ob animals; Veh-WT, wildtype C57BL/6 mice injected with saline and pair-fed to match food intake of FGF1-ob animals; FGF1-depleted, neighborhoods in which the abundance of FGF1-ob cells is decreased relative to Veh-ob cells; FGF1-enriched, neighborhoods in which the abundance of FGF1-ob cells is increased relative to Veh-ob cells; WT-depleted, neighborhoods in which the abundance of WT cells is decreased relative to Veh-ob cells; WT-enriched, neighborhoods in which the abundance of WT cells is increased relative to Veh-ob cells; rescued-depleted, neighborhoods which register a significant differential abundance of FGF1-depleted and WT-depleted; rescued-enriched, neighborhoods that register a significant differential abundance of FGF1-enriched and WT-enriched.


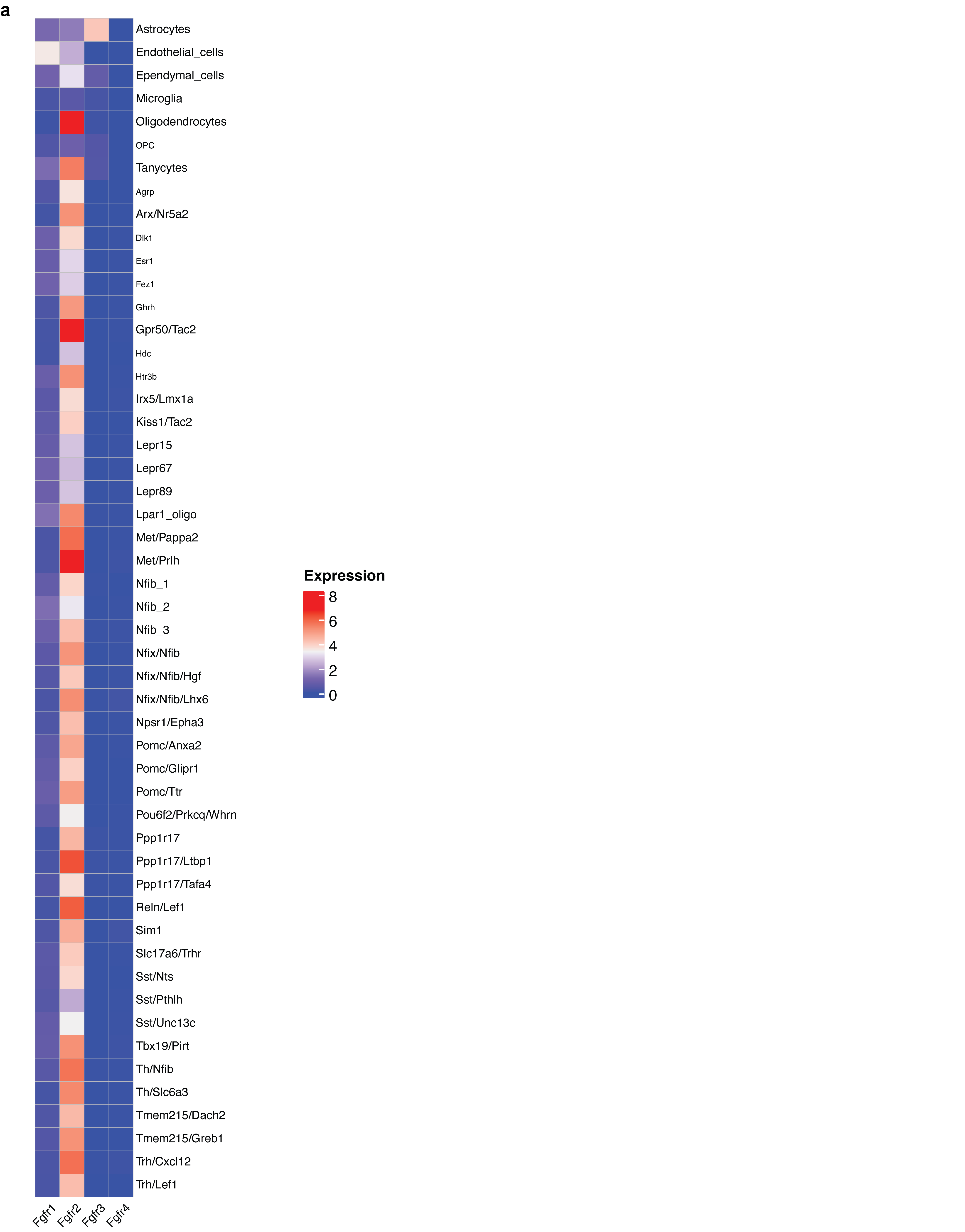


**Supplementary Figure 4**. Heatmap showing the expression of all *Fgfr* receptors across all neuronal cell types.


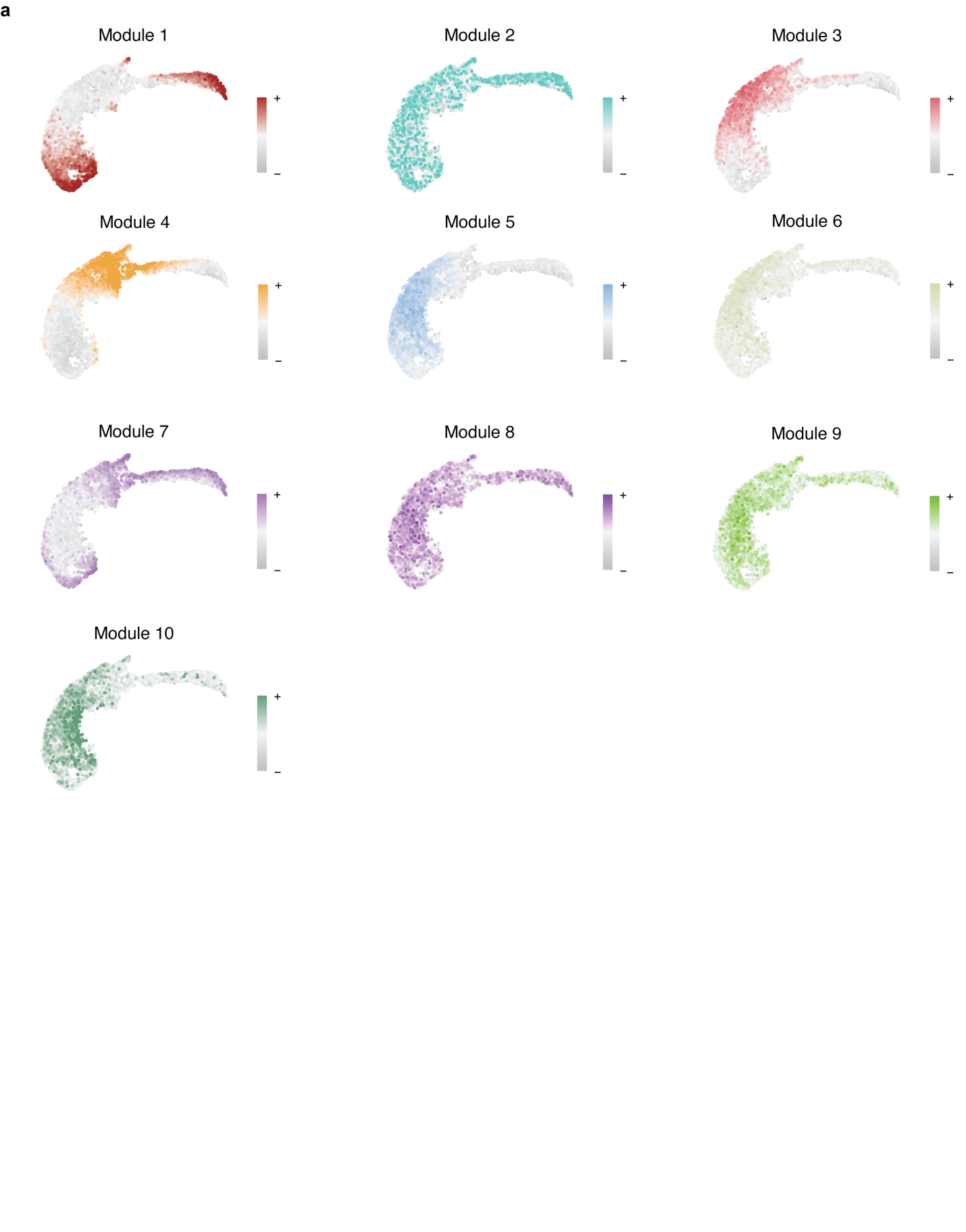


**Supplementary Figure 5**. **a**, UMAP feature plots showing the harmonized module eigengene expression across all 10 hdWGCNA-defined modules in AgRP neurons from Veh-ob and FGF1-ob mice. Cells are color-coded by eigengene score. Module has been abbreviated to M in the main text. Abbreviations: UMAP, uniform manifold approximation and projection; FGF1, fibroblast growth factor 1; FGF1-ob, Lep^ob/ob^ mice treated with FGF1 via icv injection; Veh-ob, Lep^ob/ob^ mice injected with saline and pair-fed to match food intake of FGF1-ob animals


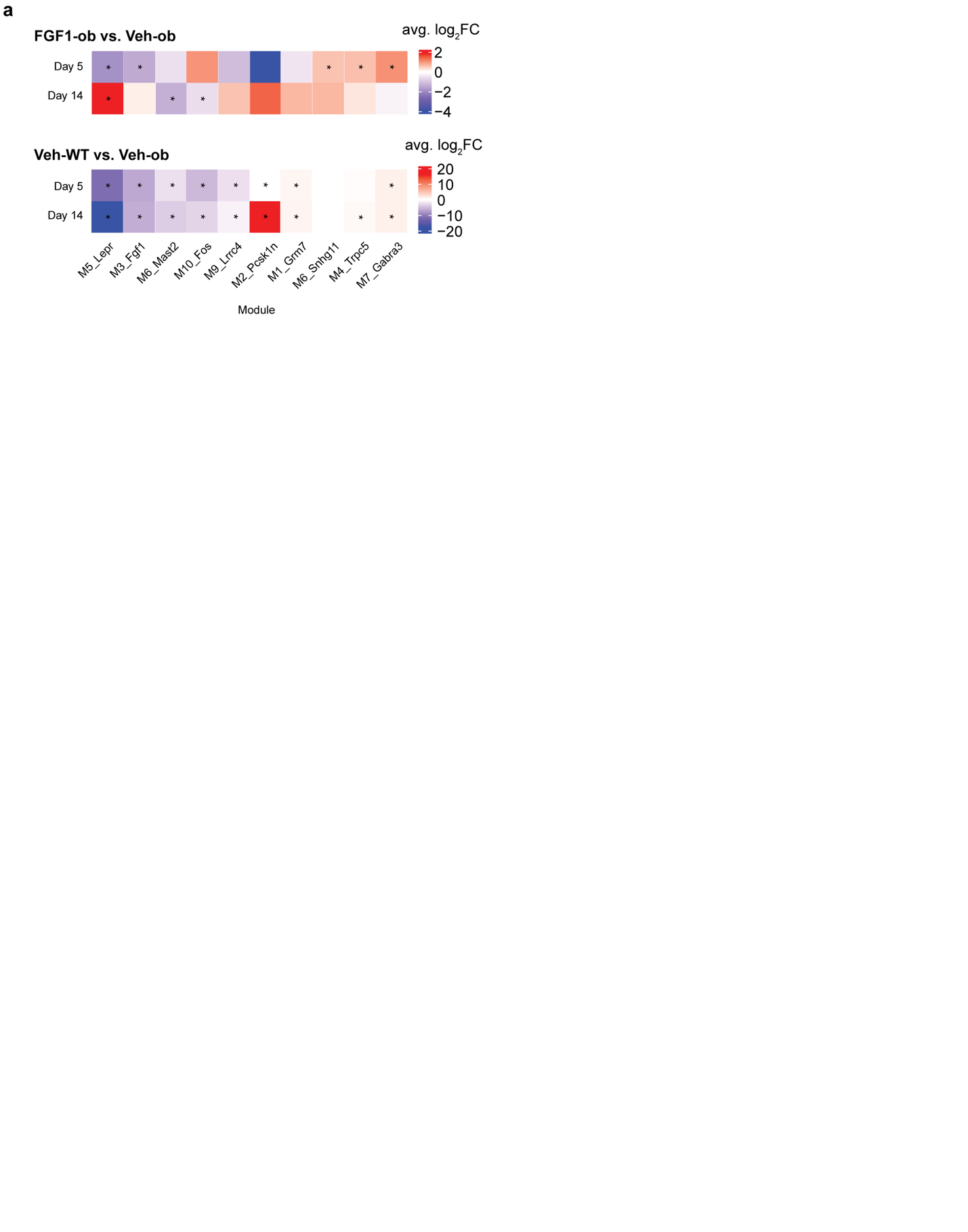


**Supplementary Figure 6.** Heatmap showing the results of differential module eigengene analysis for FGF1-ob vs. Veh-ob (left) and Veh-WT vs. Veh-ob (right) across all modules. Colors are scaled by average log2 fold-change. Significantly different modules were annotated (*, Bonferroni-adj. p<0.05).


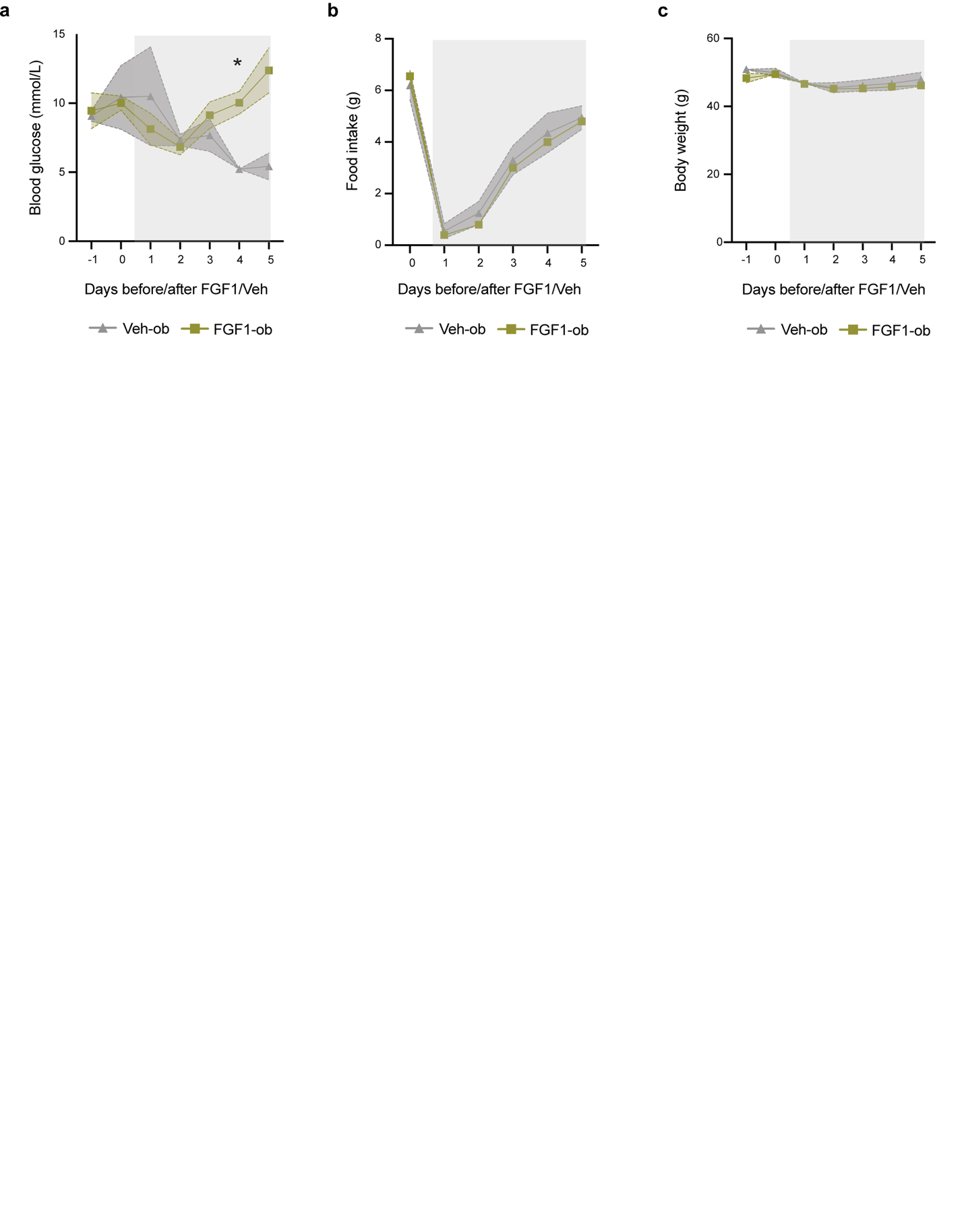


**Supplementary Figure 7. a**, Blood glucose measurements of FGF1-ob, Veh-ob, or Veh-WT-group mice sacrificed five days after injections. **b**, Food intake measurements of FGF1-ob, Veh-ob, or Veh-WT-group mice sacrificed five days after injections. **c**, Body weight measurements of FGF1-ob, Veh-ob, or Veh-WT-group mice sacrificed five after injections. Differences between groups were identified using two-way ANOVA with multiple comparisons when applicable (*, p<0.05). Abbreviations: FGF1, fibroblast growth factor 1; FGF1-ob, Lep^ob/ob^ mice treated with FGF1 via icv injection; Veh-ob, Lep^ob/ob^ mice injected with saline and pair-fed to match food intake of FGF1-ob animals; Veh-WT, wildtype C57BL/6 mice injected with saline and pair-fed to match food intake of FGF1-ob animals; ANOVA, analysis of variance


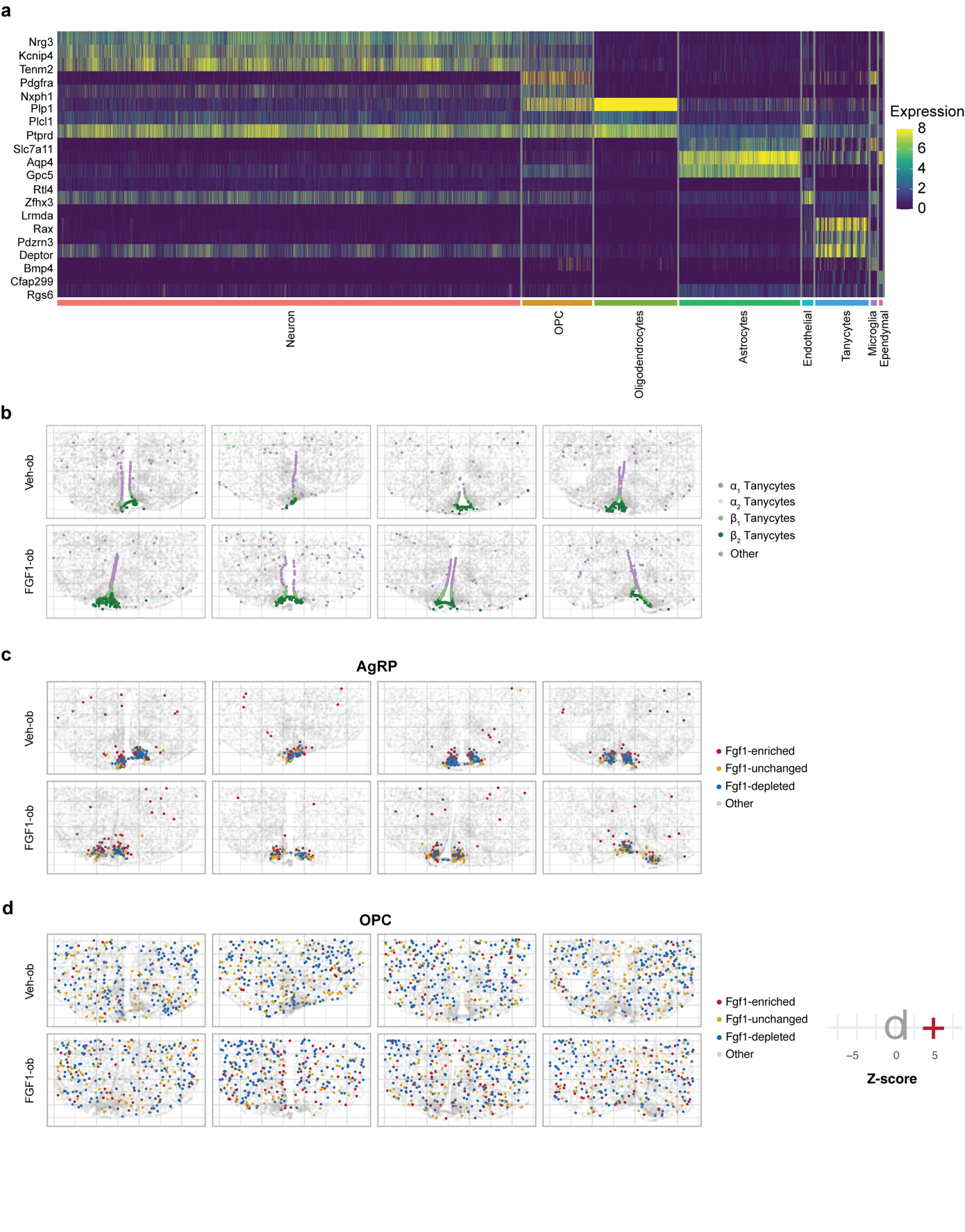


**Supplementary Figure 8. a**, Heatmap of all genes across all cells. Illustrates how spatial resolution uncovers marker genes of the main cell populations. Color gradient reflects relative expression levels. **b,** Spatial plot showing the bottom of the third ventricle in the hypothalamus. Tanycyte sub-populations are color-colored accordingly. **c,** Spatial plot highlighting the spatial position and polarity of AgRP neurons. **d,** Spatial plot highlighting the spatial position and polarity of OPC glia cells (left). Differential abundance analysis of FGF1-ob vs Veh-ob at day 5 using Molecular Cartography (right). "O" denotes an overall significant difference in cell abundance; "|" indicates differential counts between negative and neutral for specific cell types; "+" highlights differential counts between positive and neutral for specific cell types. Abbreviations: FGF1, fibroblast growth factor 1; FGF1-ob, Lep^ob/ob^ mice treated with FGF1 via icv injection; Veh-ob, Lep^ob/ob^ mice injected with saline and pair-fed to match food intake of FGF1-ob animals


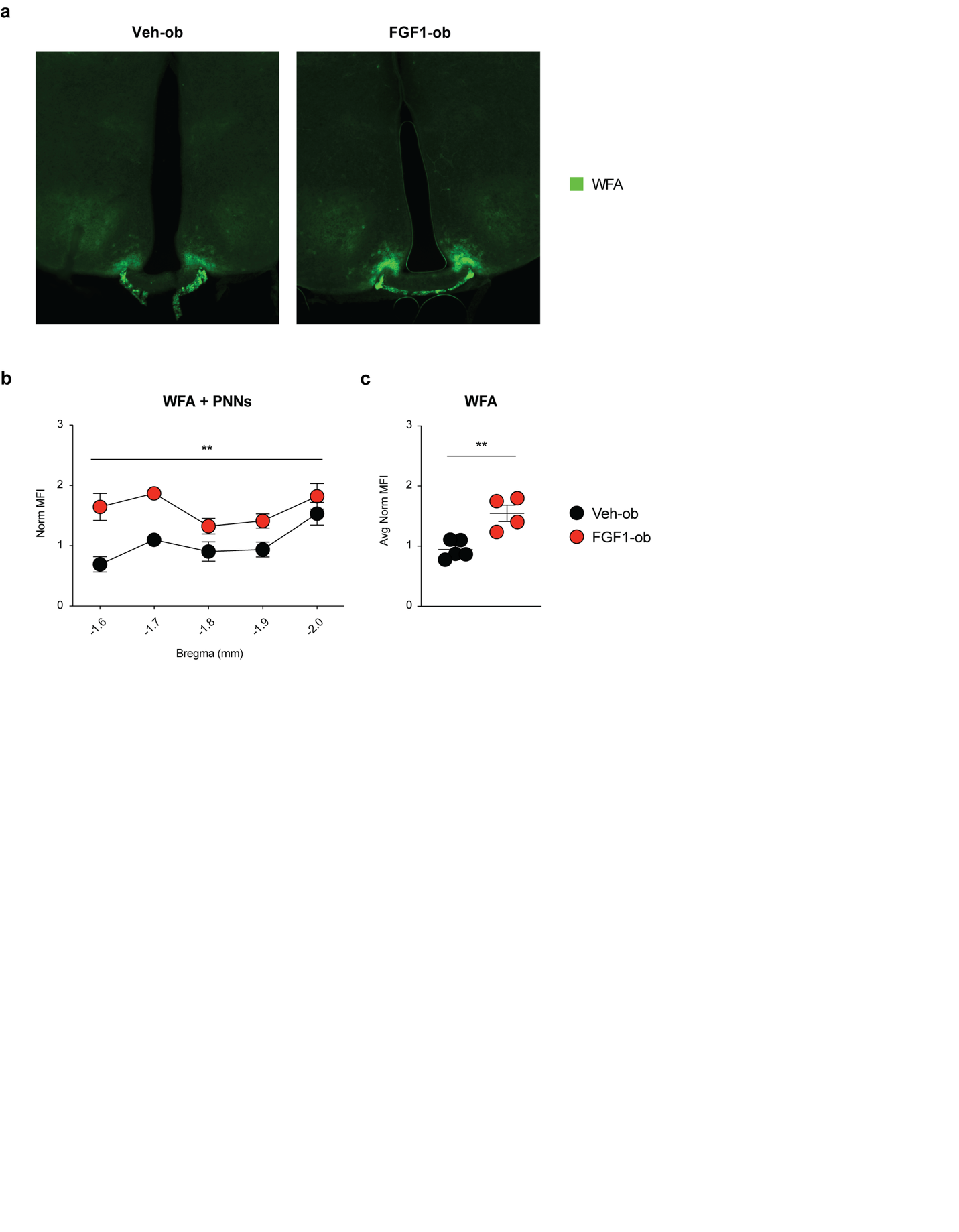


**Supplementary Figure 9. a,** Intracerebroventricular injection of FGF1 (3 ug) targeting the lateral ventricle in adult, male Lep*^ob/ob^* mice showed significant increase. **b,c** WFA+ PNN CS/DS-GAG mean fluorescent intensity (MFI) throughout the rostral-caudal extent of the arcuate nucleus, averaged in (c). Stats: n=5 Veh-ob and n=4 FGF1-ob mice. Mixed effects model with matched stereology regions fit to a single model (treatment) p=0.0028. Averaged normalized MFI using unpaired t-test p=0.0038.


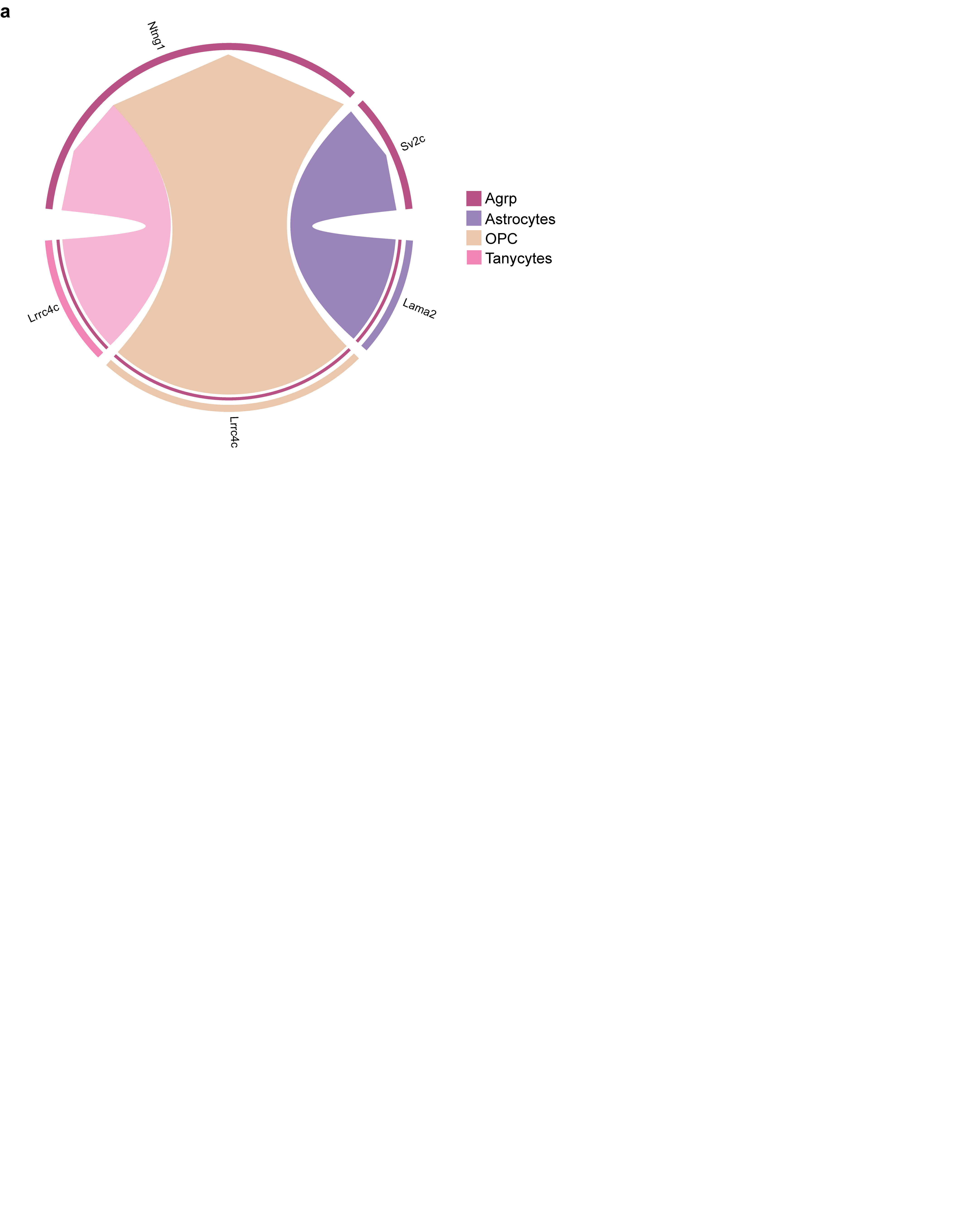


**Supplementary Figure 10. a**, Chord diagram visualizing the down-regulated cell-cell communication network of AgRP neurons and non-neuronal cells in FGF1-ob vs. Veh-ob mice. Abbreviations: FGF1, fibroblast growth factor 1; FGF1-ob, Lep^ob/ob^ mice treated with FGF1 via icv injection; Veh-ob, Lep^ob/ob^ mice injected with saline and pair-fed to match food intake of FGF1-ob animals
