# Supplementary material for: Sustained diabetes remission induced by FGF1 involves a shift in transcriptionally distinct AgRP neuron subpopulations
Source: Mol Metab. 2025 Dec 9;103:102300. doi: 10.1016/j.molmet.2025.102300 (PMC12808567; doi:10.1016/j.molmet.2025.102300)
Supplement: Multimedia component 3 [file mmc3.docx]

# Supplementary Note 1

**Gene panel selection.** We selected a panel of 100 genes compatible with the limits of the Molecular Cartography (MC) technology. Because this selection was carried out at an earlier point in time, this analysis relied on pseudocounts generated by Salmon Alevin and Alevin-fry.

Specifically in the case of this analysis, data from each snRNA-seq run was normalized using SCTransform with the glmGamPoi method and Illumina flowcell as a batch to be regressed out. Integration features were selected (nfeats=5000) and used to prepare the datasets for integration. PPCA was performed on each dataset with 30 dimensions. Subsequently integration anchors were identified using the Reciprocal PCA (RPCA) method, with parameters set to dims=30 and k.anchor=25. The datasets were integrated using these anchors with a weighting parameter k.weight=100. The integrated data were then subjected to PCA, and clustering with a resolution set to 0.8 and UMAP generation using the top 30 PCs.

The snRNA-seq data was subset to only contain cells from Day 5 and ob/ob animals. Due to a 3:1 overrepresentation of neurons to non-neuronal cells, the neurons were randomly subsampled to achieve a balance between these two classes (i.e., n neurons = n other cells). As part of the gene space filtering process, only named ENSEMBL genes were retained, and the feature space was further limited to the 10,000 most informative genes by the retain_informative_genes function from the geneBasisR package. After these subsetting steps, data was preprocessed with SCTransform as described above.

An initial round of gene panel selection was carried out using the geneBasisR function gene_search with a target of 100 genes for the panel starting with the preselected genes: *Pdgfra* , *Bmp4*, *Plp1* , *Aqp4*, *Rax*, *Agrp*, *Pomc*, *Htr3b*, *Lef1*, and *Lmx1a*.

In analyzing the resulting gene panel, it was noted that the geneBasisR evaluate_library function metrics for cell label accuracy and cell-cell distance in PCA space reached a saturation point for neurons at about 85 genes. Consequently, the final 15 genes were discarded. The selection was repeated with the 85 previously selected genes only using the non-neuronal cells in order to enhance performance in this subset. The resulting gene list is included in the Supplement and also available from Resolve Biosciences under ID: K7W1E.

**Glia marker genes for the spatial transcriptomics experiment**. Gene markers were defined explicitly for astrocytes (expressed: *Gfap, Aldh1l1, Aqp4, Bmpr1b, Fgfr3*), oligodendrocytes (expressed: *Opalin, Plp1*; not expressed: *Rbfox3*), OPCs (expressed: *Olig2, Olig1, Vcan, Gpr17*), endothelial cells (expressed: Cldn5, Ftl1, Pecam1, Dcn; not expressed: Rbfox3, Snap25), microglia (expressed: *Cx3cr1, Ikzf1*), tanycytes (expressed: *Rax, Col23a1, Crym*; not expressed: *Rbfox3*), VLMCs (expressed: *Lum, Col1a1, Dcn*), pericytes (expressed: *Pdgfrb*; not expressed: *Col1a1*), ependymal cells (expressed: *Foxj1, Pifo, Dynlrb2*), and neurons (expressed: *Rbfox3, Snap25*; not expressed: *Cx3cr1, Ikzf*1). In this case, cells which were identified as neurons were dropped. In the spatial transcriptomics analyses, tanycytes were further broken down into subtypes using predefined marker genes as follows: α1 Tanycytes (expressed: *Mafb, Necab2, Slc17a8*), α2 Tanycytes (expressed: *Pdzph1*), β1 Tanycytes (expressed: *Frzb*), and β2 Tanycytes (expressed: *Scn7a, Adm*).

# Supplementary Note 2

**Labeling cells based on their shared neighborhood labels**. As described previously, whenever Milo is presented a set of cells it samples that population repeatedly to form multiple overlapping neighborhoods. Following differential abundance testing, each neighborhood receives a label (e.g. - “enriched” for ‘FGF1-ob vs. Veh-ob’ or “rescued-enriched” when ‘Veh-WT vs. Veh-ob’ is also “enriched”). To facilitate an equitable representation of less frequent labels and mitigate the potential dominance of more prevalent ones, labels were reassigned to cells based on a weighted voting mechanism. In this context, the following definitions apply:

- Let $N$ be the set of all neighborhoods
- Let $C$ be the set of all cells
- Let $L$ be the set of all labels
- $freq\left( l \right)$ is the frequency of the label $l$ among all neighborhoods with a range of $\left[ 0,1 \right]$
- $w\left( l,n \right)$ is the weight of label $l$ in the neighborhood $n$, calculated as $w\left( l,n \right)=1-freq\left( l \right)$, otherwise $w\left( l,n \right)=0$
- $N_{c}$ is the set of a neighborhoods which include cell $c$

The score for a label l on cell c is given by

- $S\left( c, l \right) = \sum_{n\epsilon N_{c}} w\left( l,n \right)$

The label assigned to each cell is the one with the highest score:

- $label(c)= argmax S\left( c, l \right)$

Under this setup, each neighborhood in which a cell appears casts a vote for its label, with the weight of each vote inversely proportional to the label's overall frequency across all neighborhoods. The weighted voting strategy reduces the influence of common labels, thereby preserving the diversity of less common labels in the dataset. For each cell, the label associated with the highest cumulative weight from all neighborhoods to which the cell belonged was assigned as its final label. This method ensures a balanced label distribution within cells reflecting the label distribution of neighborhoods.

Combined Neighborhood State Labels Based on differential abundance Changes in ob/ob and BL6 Animals

| **FGF1-ob vs. Veh-ob** | **Veh-WT vs. Veh-ob** | **combined label** | **in this neighborhood:** |
| --- | --- | --- | --- |
| enriched | enriched | rescued-enriched | DA increase of icv FGF1-ob vs Veh-ob cells  AND  DA increase of wild-type vs Veh-ob cells |
| depleted | depleted | rescued-depleted | DA decrease of icv FGF1-ob vs Veh-ob cells  AND  DA decrease of wild-type vs Veh-ob cells |
| enriched | unchanged | FGF1-enriched | DA increase of icv FGF1-ob vs Veh-ob cells |
| depleted | unchanged | FGF1-depleted | DA decrease of icv FGF1-ob vs Veh-ob cells |
| unchanged | unchanged | unchanged | No change. |
| unchanged | enriched | WT-enriched | DA increase of wild-type vs Veh-ob cells |
| unchanged | depleted | WT-depleted | DA decrease of wild-type vs Veh-ob cells |
| enriched | depleted | away-enriched  (very rare outcome) | DA increase of icv FGF1-ob vs Veh-ob cells  AND  DA decrease of wild-type vs Veh-ob cells |
| depleted | enriched | away-depleted  (very rare outcome) | DA decrease of icv FGF1-ob vs Veh-ob cells  AND  DA increase of wild-type vs Veh-ob cells |
